# Greener and whiter analytical method development and validation for determining the presence of zolpidem tartrate infused in apple juice using RP-HPLC *via* magnetic solid-phase extraction followed by LC-MS confirmatory analysis†

**DOI:** 10.1039/d4ra04303k

**Published:** 2024-09-04

**Authors:** Revathy Sundara Moorthy, G. Swetha, Rohini Rondla, Anren Hu, Narmada Vallakeerthi, P. Muralidhar Reddy

**Affiliations:** a Department of Chemistry, University College of Science, Osmania University Tarnaka Hyderabad Telangana 500007 India pmdreddy@gmai.com; b Department of Chemistry (H & S), Vidya Jyothi Institute of Technology Aziz Nagar Gate Hyderabad Telangana 500075 India; c Department of Laboratory Medicine and Biotechnology, Tzu Chi University Haulien 97004 Taiwan; d Department of Pharmacy, University College of Technology, Osmania University Hyderabad 500007 Telangana India

## Abstract

The research work entails a newly developed RP-HPLC method, aimed at analyzing the modern date rape drug, zolpidem tartrate (ZT), infused in apple juice matrix. The work relies on dispersive solid-phase extraction (DSPE) with polyethylene imine (PEI)-coated magnetic nanoparticles to preconcentrate zolpidem from the matrix, in the presence of trifluoroacetic acid (TFA) for matrix isolation, for the first time. The optimized conditions emphasize the use of an environmentally preferable mobile phase [methanol: 0.5% acetic acid (60 : 40% v/v; pH 2.50)] at a 1 ml min^−1^ flow rate, employed with a Platisil Octa-Decyl Silane (ODS) column (250 × 4.6 mm; 5 μm). Further, the validated results were confirmed to be within the ICH guidelines, marking the method demonstrated to be linear (*R*^2^ = 0.9988; 0.9957), robust (% RSD below 1), sensitive (LOD = 1.8 μg ml; LOQ = 6 μg ml^−1^), precise and accurate (% recovery = 92–120%). Following the same conditions, a confirmatory analysis of zolpidem was accomplished using LC-MS, verifying the method's suitability notably, with good peak resolution, less matrix interference and a confirmation of the presence of zolpidem using mass spectrometry. The recycling ability of the PEI@SiO_2_@Fe_3_O_4_ nanoparticles was also assessed. To determine the sustainability of the proposed work, a greener and whiter assessment has been carried out in a comparative mode with previous similar works. For green tools, the recently developed AGREE software was utilized for assessing the method's greeness and it demonstrated a good green score of 0.68, supported by method assessment using ComplexGAPI software. For the assessment of the method's blue principles, the latest software utilizing the blue applicability grade index (BAGI) was applied, resulting in a decent score of 62.5. To consider sustainability, the RGB methodical software in its latest version the RGBfast model, was incorporated in the study for furnishing a balance of the three different major principles (Red–Green–Blue) and for assessing a check on sustainability of the current method compared to similar previously established proposed works.

## Abstract 2

The research work entails a newly developed RP-HPLC method, aimedat analyzing the modern date rape drug, zolpidem tartrate (ZT), infused in apple juice matrix.

## Graphical abstract

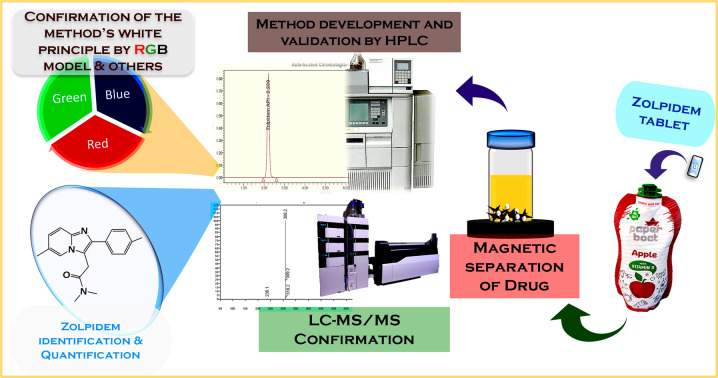

## 1. Introduction

Recently, a gradual shift has been observed in the medical field related to the move away from the use of common and highly sedative drugs to new sedative hypnotics. The predominant benzodiazepines function in a non-selective way by binding to both omega-1 and -2 receptor sites but pose more side effects and a greater risk of dependence. Hence, to reduce drug abuse, side effects, and dependence on these drugs, a new class of drugs, *i.e.*, non-benzodiazepines, especially Z-drugs, has been developed and brought to the medical market.^1–4^ Among the non-benzodiazepines, an imidazopyridine derivative, namely zolpidem (ZM), has attracted much attention over others in consideration of its selective interaction with the omega-1 receptor alone.^5,6^ In spite of their medical benefits, their various properties, such as quick action together with causing hallucinations and anterograde amnesia, have seen increasing misuse, particularly triggering their use in drug-facilitated crimes and more seldom in sexual assault cases.^7,8^ Zolpidem, as the most prescribed antipsychotic medication, has become a key focus due to its increasing misuse as criminals can easily slip the zolpidem into beverages to allow them to perpetrate various crimes, including robbery, drug-facilitated sexual assault (DFSA), and kidnapping.^9–11^ A temporal study has also been reported on zolpidem medication towards the risk of suicide.^12^ In view of the recent concerns over zolpidem-associated crimes, zolpidem has become a key focus of study.

The previous literature includes reports on the extraction, method development, and determination of zolpidem tartrate (ZT) and other similar drugs in different biological fluids^13–18^ and hair.^19–23^ Further reports have focused on the detection of the most common narcotics and sedatives^24^ in food matrices, like chocolate, cream biscuits, cold drinks,^25–28^ and water samples.^29^ Some recent comparable works include one on patulin contaminant identification in apple juice *via* LLE extraction using an LC-MS/MS analytical method,^30^ while another reported a method for ZT detection in human plasma *via* a dispersive liquid microextraction method with HPLC-PDA (photodiode array).^31^ Though there are similar classes of works, there are no reports of zolpidem detection in edible matrices. Hence, to the best of our knowledge, the current work is new and was performed for the first time in the field of analytical chemistry.

To develop greener yet efficient economical extraction procedures, solid-phase extraction (SPE) has drawn favorable attention among researchers, notably in the analytical field. Among the different SPE methods, dispersive solid-phase extraction (DSPE), especially using magnetic nanoparticles (MNPs) as in magnetite solid-phase extraction (MSPE), has been proven to be beneficial due to its simple separation process. The isolation principle works *via* various physicochemical interactions, such as electrostatic force, hydrogen bonding, π–π interactions, and covalent or ionic bonding. In consideration with this principle, the surface modification of MSPE can be done depending on the type of analytes considered for isolation.^32^ The rationale for choosing MSPE is that it can reduce the typical large solvent usage, matrix interferences, time consumption, and can replace centrifugation by magnetic isolation;^33,34^ thereby, reducing the time, energy, and labor needed, while also being more specific as well as recyclable.^35,36^ For efficiently precipitating proteins, sugars, and carbs, TFA is a popular choice in extraction as it is strongly acidic in nature, due to the presence of the highly electronegative fluorine in its structure.^37–39^ Various literature works have reported the use of MNPs. For instance, Reddy P. M. *et al.* reported the use of PEI@SiO_2_@Fe_3_O_4_ nanoparticles (NPs) in capturing Gram-positive and Gram-negative bacteria with good efficiency and a detection limit of 10^4^ CFU ml^−1^.^40^

The current study concerns a new magnetic dispersive solid-phase extraction procedure, preceded by method optimization and validation of the presence of zolpidem tartrate (ZT) infused in apple juice, with the aid of reverse phase high-performance liquid chromatography (RP-HPLC). To prove the sustainability of the newly developed method, a green and white assessment was carried out.

Although in DFC and DFSA crimes, biological samples play a crucial role in identification, there remains a constraint of its use to within a specified period of time. It is not feasible all the time to work with biological samples, as gaps or delays in reporting, collection, and analysis may become an obstacle to accurate results and identification.^41,42^ There is also a growing need to analyze for drugs in food drinks used in crimes. Though there are many cases reported of the use of date rape drugs or other narcotic-related drugs in food and drink matrices, the analysis of such matrices has received limited attention in the research field. Cold drinks and fruit juices are some of the possible matrices that carry a risk of the intentional infusion of drugs.^43–46^ In the present study, as a common and easily available flavor and drink, apple juice was chosen for study. Despite zolpidem-infused apple juice cases not being specifically reported, forensic cases are always unanticipated, providing new challenges every time. Hence the method can also be applicable for similar matrices too.

In order to achieve an environmentally friendly method, recent green software, such as the Complementary Green Analytical Procedure Index (Complex-GAPI)^47^ and Analytical GREEnness metric approach (AGREE),^48^ have been developed to create awareness about solvent consumption and the nature of the resources used as well as to caution about the use of toxic reagents. Further, white assessments are now also gaining ground in analytical chemistry. This method additionally incorporates a “red” evaluation for proving the reliability of a proposed method, and “blue” evaluation depicting the practicality of a method besides a “green” evaluation. The assessment can be done by using the Red–Green–Blue (RGB) fast model,^49^ a latest version of the RGB model with advancements. In addition, very recent blue assessment software utilizing the blue applicability grade index (BAGI)^50^ has been developed. There are few recent reports on comparative studies including green and white assessments of different methodologies for the same and similar drugs.^51,52^ Further, these comparative studies could aid in the easy differentiation of suggested work to previously reported ones, by providing each method's greenness, analytical performance, and their practicality for comparison. A comparative study was also performed for the present work, and the findings suggest that the current work abides by many parameters matching the green, red, and blue principles.

In view of its sensitivity, simplicity, easy availability, economic nature, and moderate energy consumption, HPLC^53,54^ was chosen for the method development and validation in the current work. To make the study more valid, a confirmatory analysis was performed using LC-MS.^55^ This offers certain advantages, such as better resolution, ability for structural elucidation, and effective elemental analysis, allowing an accurate qualitative assessment of the presence of zolpidem through extraction under newly developed conditions. Further, by accounting the instrument's short availability, high energy and cost consumption the LC-MS was applied for confirmatory analysis and not for method development, while method development and validation was performed through RP-HPLC.^56^

## 2. Experimental section

### 2.1. Chemicals used

The chemicals used were generally reagent grade as obtained from commercial sources. Zolfresh^®^10 tablets with 10 mg of zolpidem tartrate were acquired from Abbott (Himachal Pradesh, India). The iron salts ferric chloride hexahydrate with a purity of 98% and ferrous chloride with a purity of 98.5% were purchased from Molychem (Mumbai, India) and Avra (Mumbai, India), respectively. Ammonia solution 0.91 d with 25% purity was acquired from Finar (Ahmedabad, India). APTES (3-aminopropyl-triethoxysilane) reagent with a purity of 98% was purchased from Spectrochem. All the solvents used, such as ethanol with 98% purity purchased from TCI (Hyderabad, India) and toluene, were of analytical grade. The reagents polyethylene imine (PEI) and tetraethyl orthosilicate (TEOS) with a purity of 98% were purchased from TCI (Hyderabad, India). Hydrochloric acid (HCl) with 32% purity was purchased from Avra Chemicals. HPLC-grade methanol with a purity of ≥99.9% was obtained from Honeywell (New Delhi, India) and water was from Finar (Ahmedabad, India) with ≥99.9% purity. Trifluoroacetic acid (TFA) with a purity of 99% was purchased from Sisco Research Laboratory (New Mumbai, India). Acetic acid (Glacial) (≥99.7% purity) was procured from Qualigens (Mumbai, India). Apple juice (Paper boat) (Haryana, India) for the matrix was purchased from the supermarket.

### 2.2. Instrumentation

High-performance liquid chromatography (HPLC) was performed using a Waters Alliance 2690 HPLC system (USA) equipped with a Waters 996 photodiode array (PDA) detector. Ultraviolet-visible spectroscopy (UV-vis) was used for the preliminary analysis using a Shimadzu-UV 2600 (Japan) system. Fourier transform infrared spectrophotometry (FTIR) was performed on a Shimadzu – IRPrestige-21 system (Japan). X-Ray diffraction (XRD) was performed on a Rigaku Smart Lab system equipped with SmartLab Studio II software (Japan). Scanning electron microscopy and energy-dispersive X-ray analysis (SEM-EDX) was carried out on a Zeiss EVO LS15 instrument equipped with a field emission source (Germany). Transmission electron microscopy (TEM) was performed with a Tecnai 12 system (FEI, Netherlands). Zeta analysis was performed with a Beckman Coulter's Delsa Max Pro system (USA).

### 2.3. Synthesis of Fe_3_O_4_ nanoparticles and functionalization with PEI-coated Fe_3_O_4_ nanoparticles

The Fe_3_O_4_ nanoparticles were synthesized through a co-precipitation method as reported in our previous literature. The synthesis procedure has already been used by our research group with few modifications. In brief, ferric and ferrous salts were dissolved in HCl in a round-bottom flask and placed under a nitrogen gas set-up, to maintain an inert atmosphere, resulting in an orangish brown precipitate. The reaction was performed at room temperature under stirring using a magnetic stirrer for 6 h. Subsequently, the Fe_3_O_4_ NPs synthesis was initiated *via* the addition of 25% aqueous NH_3_ solution, as confirmed by the black coloration of the resultant solution.^57^

The silica coating was obtained by re-suspending Fe_3_O_4_ NPs in 200 ml ethanol and adding 5 ml TEOS reagent and then refluxed. Further, SiO_2_@Fe_3_O_4_ NPs were re-suspended in 100 ml double-distilled water and stirred gently for about 8 h upon the addition of PEI reagent as reported, to obtain PEI@SiO_2_@Fe_3_O_4_ NPs.^31,58^ In order to obtain APTES@Fe_3_O_4_ NPs, reflux with methanol and toluene at a ratio of 30 : 70 was performed by re-suspending the Fe_3_O_4_ NPs. The reflux was performed at 110 °C along with 5 ml of APTES reagent. After 20 h of reflux, the resulting suspension was washed and dried in a hot air oven.

### 2.4. Preliminary examination

#### 2.4.1. UV analysis

A stock solution of 0.25 mg ml^−1^ ZT was taken as the standard for UV analysis to identify the *λ*_max_. About 3 ml of the ZT stock solution was taken into four different 5 ml vials. In each vial, 2 mg of four prepared MNPs, *i.e.*, core Fe_3_O_4_, SiO_2_@Fe_3_O_4_, APTES@Fe_3_O_4_, and PEI@SiO_2_@Fe_3_O_4_ NPs were accurately weighed and added to the respective vials. The solutions were ultrasonicated for 1 min and then an external magnet was used to collect the supernatant from each vial, which was transferred into a quartz cuvette for UV analysis.

#### 2.4.2. Selection of the precipitating medium

Isolating the interfering components of the apple juice matrix during the ZT analysis is critical. Hence, in order to select the appropriate precipitating medium, different pH media at varied concentrations were tested, namely 2 N acetic acid (4.0 pH), 0.5 N NaOH (13.67 pH), 1 N NaOH (14 pH), 0.5 N HCl (0.3 pH), 1 N HCl (<0.1 pH), 0.5% TFA (1.24 pH), and 1.5% TFA (0.87 pH).

1 ml of 100 μg ml^−1^ standard solution was pipetted to 1 ml of apple juice in seven different 10 ml centrifuge tubes. Different media, *i.e.*, acetic acid, 0.5 N HCl, 1 N HCl, 0.5% TFA, 1.5% TFA, were added to the respective centrifuge tubes, followed by extraction using the PEI@SiO_2_@Fe_3_O_4_ NPs and analysis by HPLC.

### 2.5. Optimization of the PEI@SiO_2_@Fe_3_O_4_ nanoparticle concentration

#### 2.5.1. Extraction procedure/adsorption efficiency of the MNPs

**Scheme 1:**
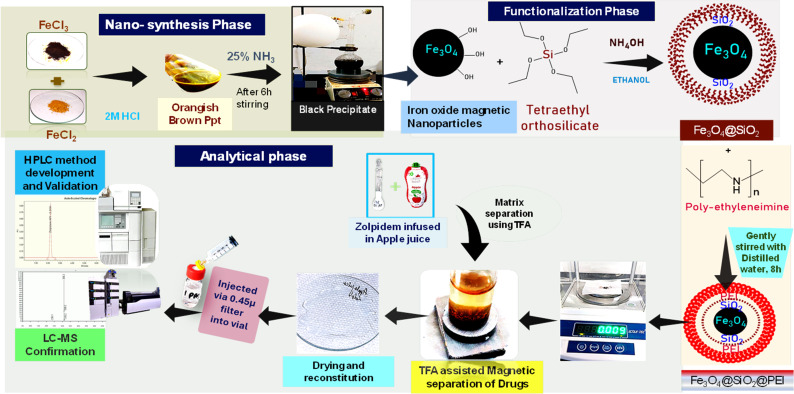
Schematic representation of the newly developed zolpidem extraction process, method development and validation by RP-HPLC.

To a 10 ml centrifuge tube, equal portions (ml) of the sample and 100 μg ml^−1^ solution of ZT API were added. The resulting solution was shaken and left for 15 min for the drug to absorb in the sample. Next, 3 ml of 1.5% TFA was added into the above solution, with shaking and then left for about 40–60 min. Later, 9 mg of PEI@ Fe_3_O_4_ NPs was added into the solution followed by ultrasonication of the NPs to ensure a thorough dispersion. An external magnet was used to hold the drug-bound NPs while the supernatant was discarded. The back extraction, *i.e.*, the desorption of ZT from the nanoparticles, was done by adding methanol. With the aid of the magnet, the supernatant was collected in a watch glass for drying in a hot air oven at ∼70–80 °C for around 15 min. The dried residue was recrystallized by using 1.5 ml of methanol and was then pipetted well around the corners of a watch glass to completely preconcentrate the ZT. Once preliminary confirmation was done, the reconstituted solution was transferred into an autosampler vial and subjected to HPLC analysis, as shown in Scheme 1.

The reusability of the PEI@SiO_2_@Fe_3_O_4_ NPs was also analyzed by UV-vis spectroscopy for the zolpidem extraction.

### 2.6. Method development

The analysis was performed with a Waters HPLC system equipped with an in-line degasser, a quaternary pump system, an autosampler, and a PDA detector. The analyte separation was accomplished using a reverse phase (RP) platisil column. According to the physiochemical properties of ZT, the maximal solubility was considered to develop a mobile phase composition. The other parameters, such as flow rate, temperature, and pH, were assessed based on the optimization tests for the method development. Finally, the optimized conditions, given below in Table 1, were applied in the further experiments for the further analysis and validation.

**Table 1:** Optimized chromatographic conditions

Column	Platisil column
	C18 octadecyl-silica (ODS)
	250 × 4.6 mm; 5 μm
Flow rate	1 ml min^−1^
Mobile phase	Solvent A – methanol
	Solvent B – 0.5% acetic acid (in water)
Gradient	Isocratic gradient – 60 : 40
Column temperature	25 °C
Injection volume	20 μl
Detector	PDA (photodiode array) – UV detector
Wavelength (*λ*_max_)	294.6 nm
Run time	10 min

### 2.7. Method validation

After the method development, the validation for the method was performed as specified by the ICH guidelines (International Conference on Harmonization of Technical Requirements for the Registration of Pharmaceuticals for Human Use)^59,60^ and Food and Drug Administration (FDA)^61^ guidelines. The following parameters with respect to validation were tested by HPLC: selectivity and specificity tests were performed using a blank, standard, and sample. Linearity assessment was performed from a concentration of 6 to 14 μg ml^−1^ in three replicas and plotted to calculate the correlation coefficient (*R*^2^).

#### 2.7.1. Accuracy

In order to assess the proximity of the expected value to that of the actual value, the standard drug at three different concentration levels was tested in three replicates, *i.e.*, by taking the mid-range of the linearity concentration of 10 μg ml^−1^ as 100%. Thereby, 50% concentration was 5 μg ml^−1^ and was prepared by pipetting 0.5 ml stock solution in to 10 ml of methanol; whereas the 150% concentration was obtained by pipetting 1.5 ml stock solution into 10 ml of methanol.

Both the limit of detection (LOD) and limit of quantification (LOQ) were calculated through the signal to noise ratio compared to the blank using the following formula:
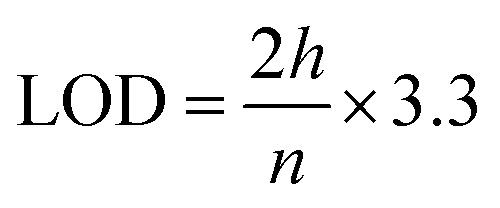

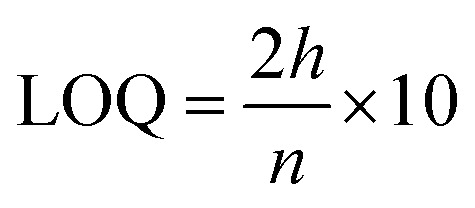
where *h* is the height of the chromatogram peak and *n* is the noise.

#### 2.7.2. Precision

A 100% homogenous concentration of the analyte was analyzed in six replicates to check the precision of the values. The same experiment was repeated on another day to examine the intermediate precision.

#### 2.7.3. Robustness

Deliberate variations from the actual optimized conditions were tested. For instance, the flow rate was changed from 1 ml min^−1^ to 1.2 ml min^−1^ (higher flow) and 0.8 ml min^−1^ (lower flow). A ±5 nm alteration was made in the wavelength from 294.6 nm to 289.6 and 299 nm. A change in the organic nature of mobile phase was also done by altering the ratio by ±10 units.

### 2.8. LC-MS confirmation

Analysis of a 10 ppm standard zolpidem tablet formulation, and the extracted zolpidem from the apple juice matrix, was performed using an Agilent 1260 Infinity II prime LC system (USA) with an MSD iQ detector for LC-MS analysis. This was done under the chromatographic conditions used for method validation with HPLC. An electrospray ionization interface (ESI) was used in the instrument, while the MS conditions are detailed in Table 2.

**Table 2:** MS conditions

Capillary current	4500–4600 Amp
Capillary voltage	3440–3460 V
Nebulizer	60 Psi
Gas temperature	350 °C
Gas flow	13 ml min^−1^
Scanning time	2 ms

## 3. Results and discussion

The work proposes the use of PEI@SiO_2_@Fe_3_O_4_ NPs for the efficient extraction of zolpidem from apple juice matrix with low matrix interference. Characterization of the PEI@SiO_2_@Fe_3_O_4_ NPs was performed to ensure the functionalization of silica, PEI, as well as to assess the physiochemical properties, using FTIR as in Table S2† and the zeta potential as in Fig. S1.† Morphological characterization was done using SEM-EDX as shown in Tables S3 and S4,† and TEM as in Table S4,† as reported in the previous literature.^40^ From the TEM, the diameter of the Fe_3_O_4_ NPs was ∼15 nm, while that of the PEI@SiO_2_@Fe_3_O_4_ NPs was ∼18 nm. For the zeta potential, there was a change observed from the negative surface charge of SiO_2_@Fe_3_O_4_ NPs to a positive surface charge, indicating the coating of PEI, as reported in the ESI in Fig. S1.†

**Fig. 1:**
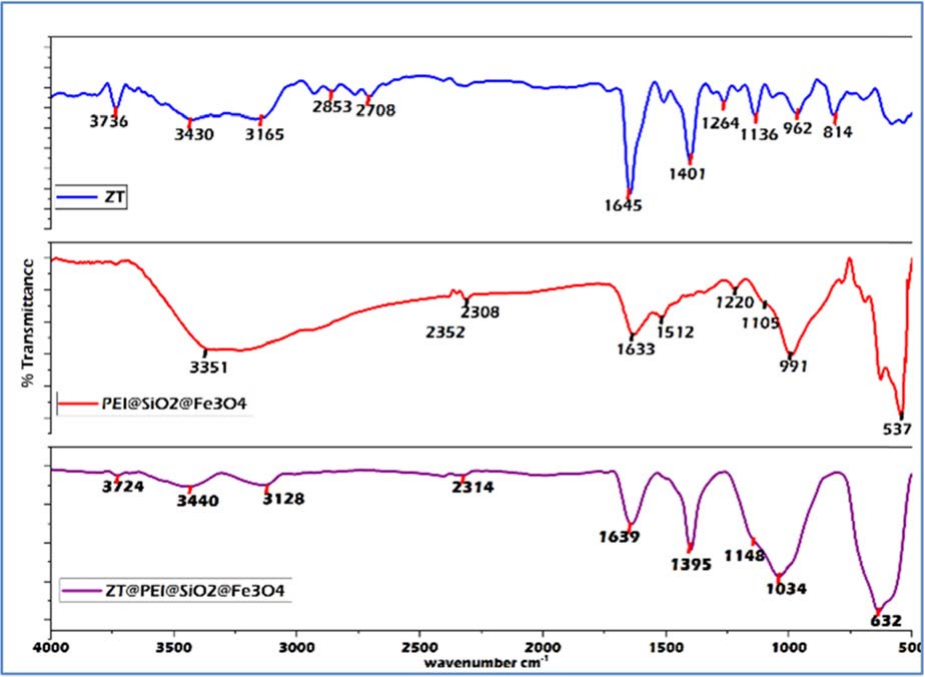
FTIR spectra of zolpidem tartrate, PEI@SiO_2_@Fe_3_O_4_, and ZT@PEI@SiO_2_@Fe_3_O_4_.

FTIR analysis was performed as shown in Fig. 1 for the pure ZT, pure PEI@SiO_2_@Fe_3_O_4_ NPs, and ZT-bound PEI@SiO_2_@Fe_3_O_4_ NPs to confirm their binding. For ZT, peaks for C–N, CH_3_, C=C, C=O, C=N, and C–H groups were observed at ∼1140, ∼1260, ∼1403; 1510, ∼1648, 1720, and ∼2709 cm^−1^, respectively. For C–N stretching related to the imidazopyridine ring, a characteristic band at ∼3135 and 3431 cm^−1^ was noticed.^62^ The PEI@SiO_2_@Fe_3_O_4_ NPs showed a characteristic band at ∼537 cm^−1^, due to the stretching vibration of Fe–O bond. For Fe_3_O_4_@SiO_2_ bonding, the vibration bands were noticed at ∼1105, 1220, and ∼991 cm^−1^ depicting the silanol group (–Si–OH), siloxane bond in the backbone (–Si–O–Si), and free silanol group, respectively.^63,64^ Additionally, apart from these, the PEI coating was confirmed by the characteristic peak at ∼1633 cm^−1^ for N–H bending, a broad band at 2847 cm^−1^ for CH_2_ asymmetric and symmetric stretching of the PEI chain, and 3351 cm^−1^ for amine group stretching,^65,66^ as shown in Fig. 1. In ZT@PEI@SiO_2_@Fe_3_O_4_ NPs, characteristic bands at ∼632, 1034, and 3440 cm^−1^ confirmed the presence of PEI@SiO_2_@Fe_3_O_4_ NPs presence, which were absent in the ZT FTIR spectrum. Further, a peak at 1638 cm^−1^ due to the presence of N–H bending was noticed for PEI@SiO_2_@Fe_3_O_4_ NPs. For ZT@PEI@SiO_2_@Fe_3_O_4_ NPs, the peaks at 1148 and 1395 cm^−1^ peak were related to C=C bonds in ZT, which were absent in the NPs, while distinctive broad peaks at 3128, 3440, and 3724 cm^−1^ were observed that were specific to imidazopyridine ring and undoubtedly confirmed the presence of ZT.

The binding of ZM to PEI@SiO_2_@Fe_3_O_4_ NPs was also evidenced by UV spectroscopy and zeta potential analysis. In the zeta potential analysis, both ZT and PEI@SiO_2_@Fe_3_O_4_ NPs separately showed positive charges but after the binding of ZT to the PEI@SiO_2_@Fe_3_O_4_ NPs, there was a change in the zeta potential charge. Additionally, RP-HPLC analysis and LC-MS proved the binding and desorption of ZT from the nanoparticles with a confirmative mass peak.

**Fig. 2:**
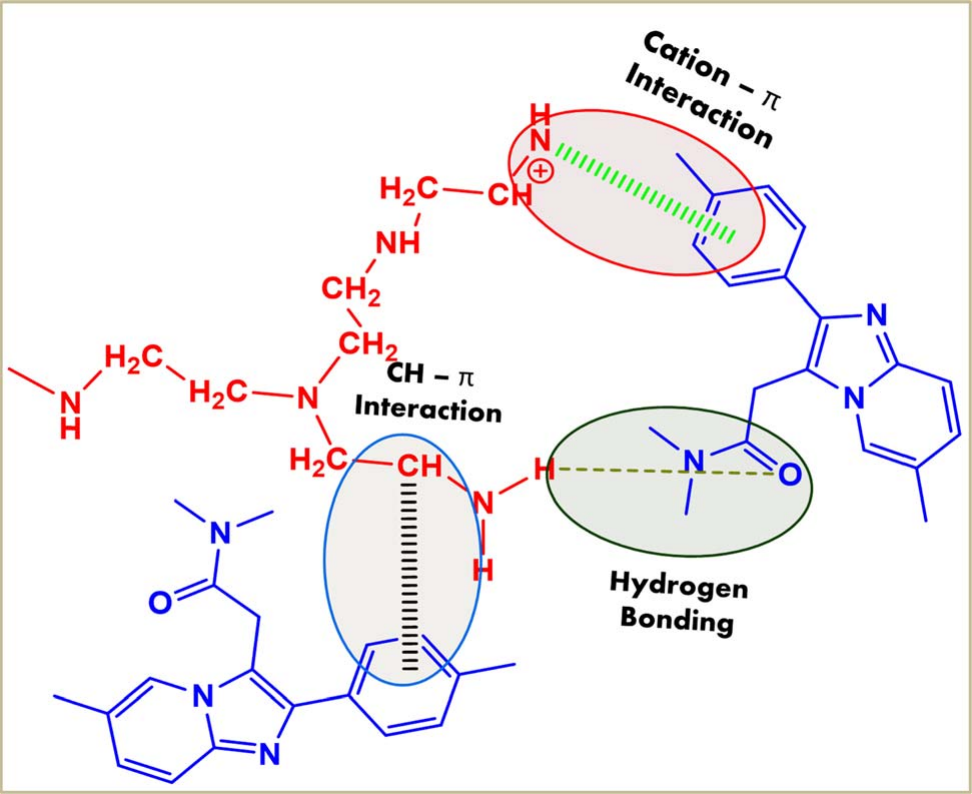
Possible mechanism of the interactions between the nanoparticles and zolpidem drug.

The possible binding mechanism involves hydrogen bonding, cation–π interactions between the NH_2_ of PEI and benzene group of ZM,^67^ and CH–π interactions between the CH_2_ group of PEI and benzene group of ZM,^68^ as illustrated in Fig. 2.

### 3.1. UV analysis

**Fig. 3:**
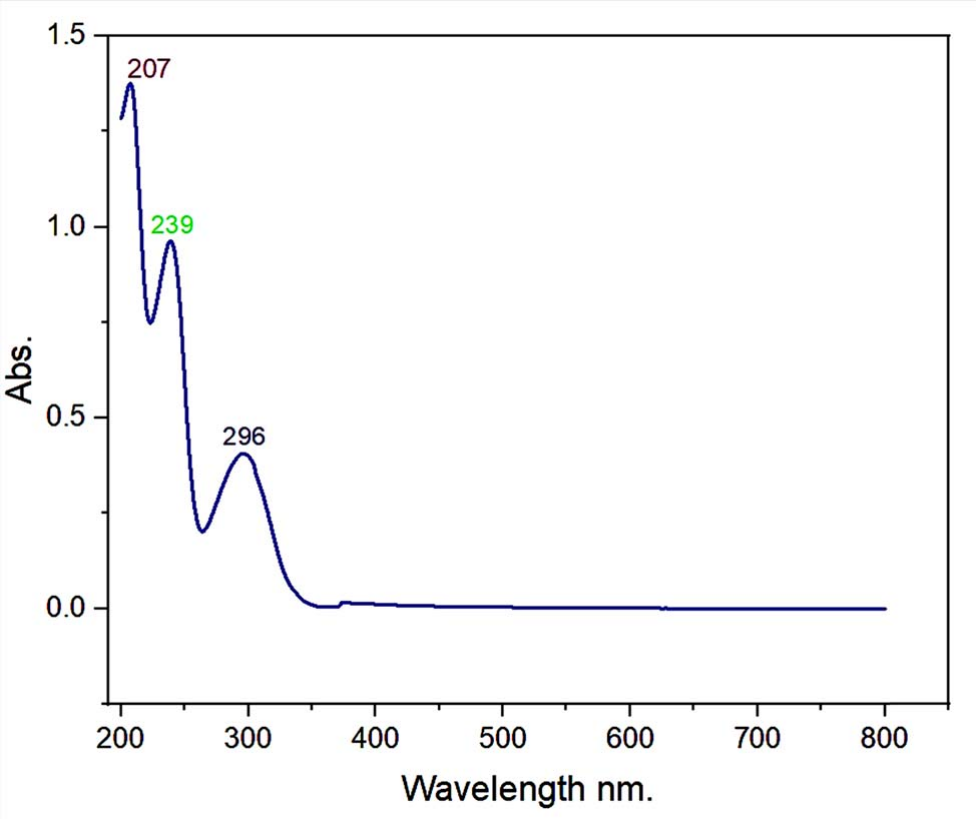
UV spectrum of pure zolpidem tartrate.

Here, 0.25 mg ml^−1^ of zolpidem was analyzed and the UV spectrum showed three maximum peaks at 207, 239 and 296 nm, as depicted in Fig. 3.

### 3.2. Optimization of MNPs

**Fig. 4:**
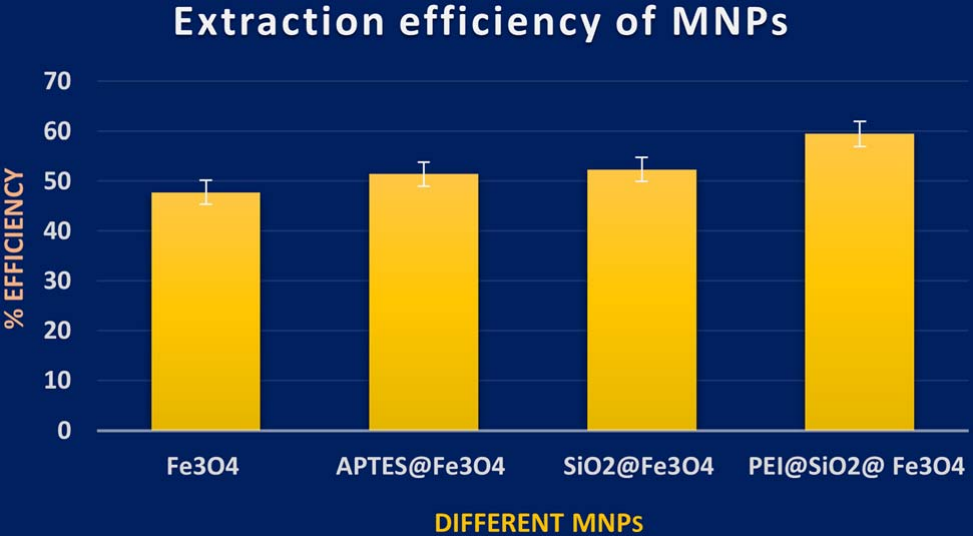
Optimization of four different nanoparticles based on their adsorption efficiency.

UV-vis spectroscopic analysis was performed for four different MNPs, in order to find the amount of ZT adsorbed by each MNP by calculating the remnant ZT concentration from the supernatant. From the analysis, among these NPs, PEI@SiO_2_@Fe_3_O_4_ NPs presented better efficiency for ZT extraction, as shown in Fig. 4. The concentration of PEI@SiO_2_@Fe_3_O_4_ NPs was also optimized at 9 mg ml^−1^ from assessing it in a range of 3–11 mg ml^−1^.

### 3.3. Optimization of the precipitating medium

**Fig. 5:**
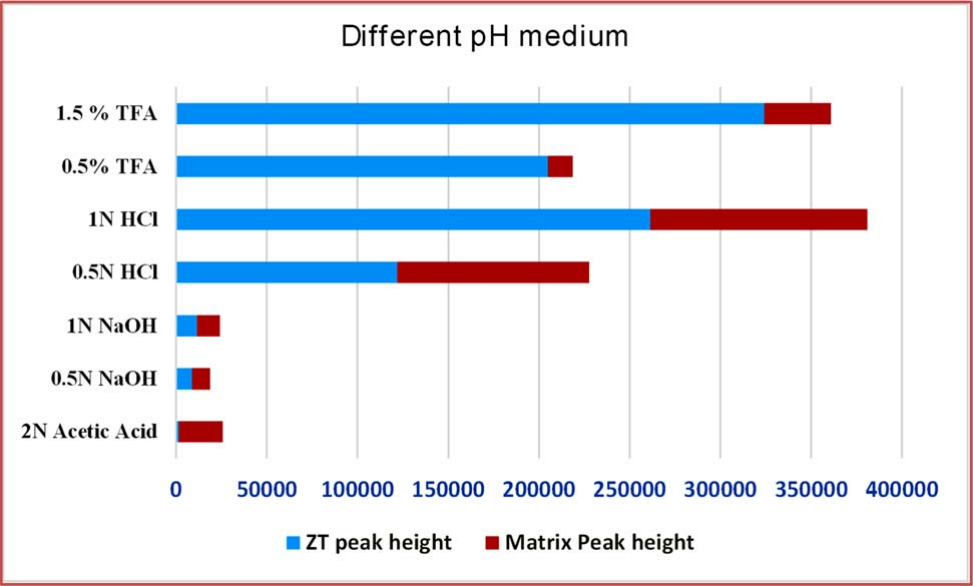
Graphical representation of the zolpidem extraction percentage and matrix effect at seven different pH media.

The extraction was carried out from apple juice with different pH media using HPLC under optimized conditions. It was proved 1.5% TFA was more efficient, and has low matrix interferences. While the other media, *i.e.*, low acidic or basic concentrations, showed very low or low extraction efficiencies, with more matrix interferences, as depicted in Fig. 5. This could be because ZT is a basic drug and can be well extracted under acidic conditions. Moreover, TFA has a good ability to break complex sugars, eventually lowering the matrix effect. Henceforth, 1.5% TFA was utilized for the extraction procedure in the following experiments.

**Fig. 6:**
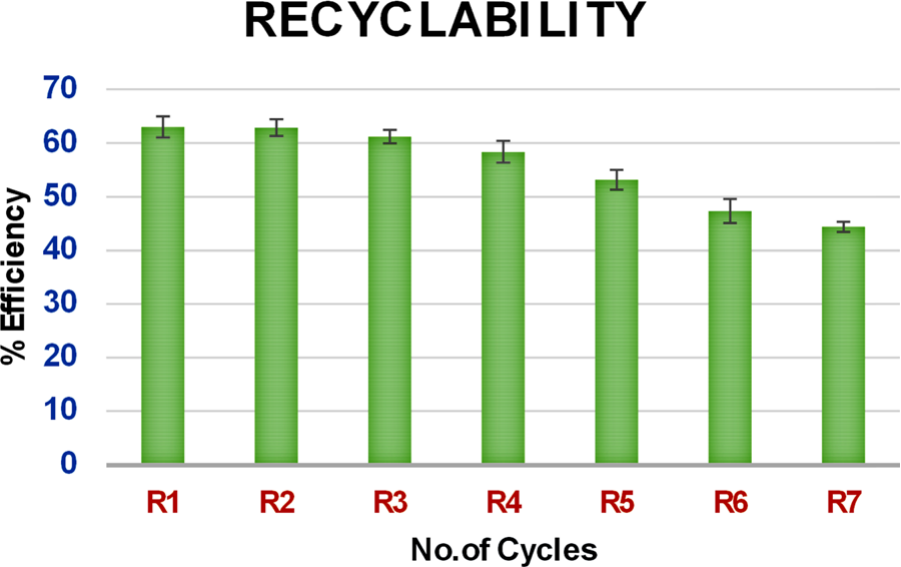
Bar graph showing the recyclability of PEI magnetic nanoparticles to extract zolpidem.

As shown in Fig. 6, on analysis of the recycling ability of PEI@SiO_2_@Fe_3_O_4_ NPs with the UV spectrum, the extraction efficiency showed a slight difference from the 4th cycle, and a marked difference from the 5th cycle with a 15% reduction.

### 3.4. Method development

**Fig. 7:**
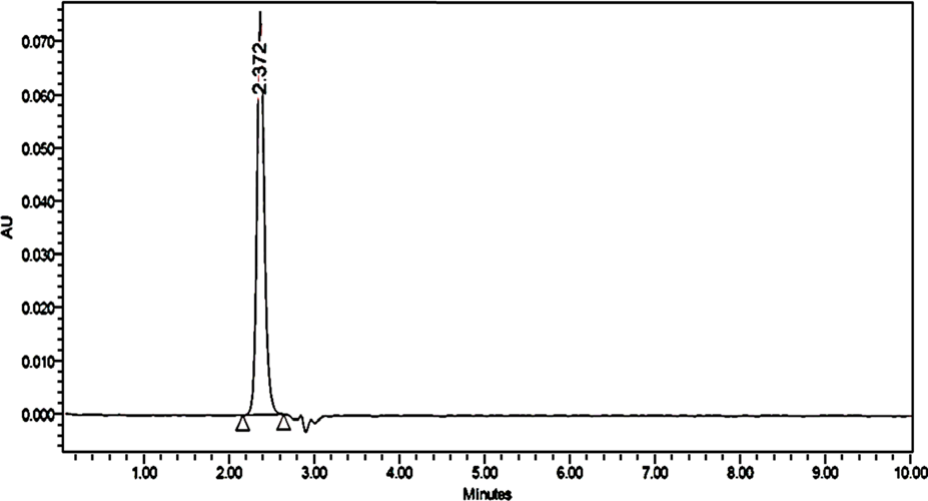
Chromatogram of zolpidem tartrate.

In several trials, a suitable condition for the analysis was tested to perform the ZT analysis, with details provided in Table 1. The run time was kept to 10 min for ±2.3 RT, in order to determine if there was any overlapping of the peaks during degradation, and the effect of matrix peaks and other impurities, if any. The data procured from the analysis were processed using Empower software. At a concentration of 10 μg ml^−1^, zolpidem in tablet form was analyzed, with the chromatogram showing the analysis was specific and selective; the system suitability parameters are given in Table 3, and the chromatograms are shown in Fig. 7.

**Table 3:** System suitable parameters of zolpidem tartrate (tablet form)

System suitability parameters	ZT results
RT	2.372
Peak area	473 665
USP plate count	3715
Tailing factor	1.14
Capacity factor (*K*)	1.554

### 3.5. Method validation

#### 3.5.1. Linearity

**Fig. 8:**
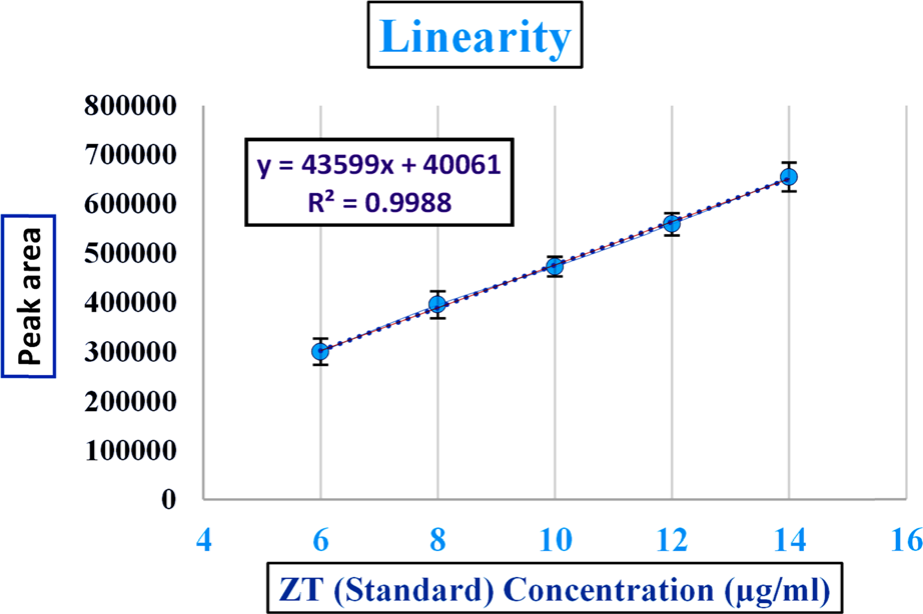
Linearity of zolpidem tablet formulation results in the 6 to 14 μg ml^−1^ concentration range.

**Fig. 9:**
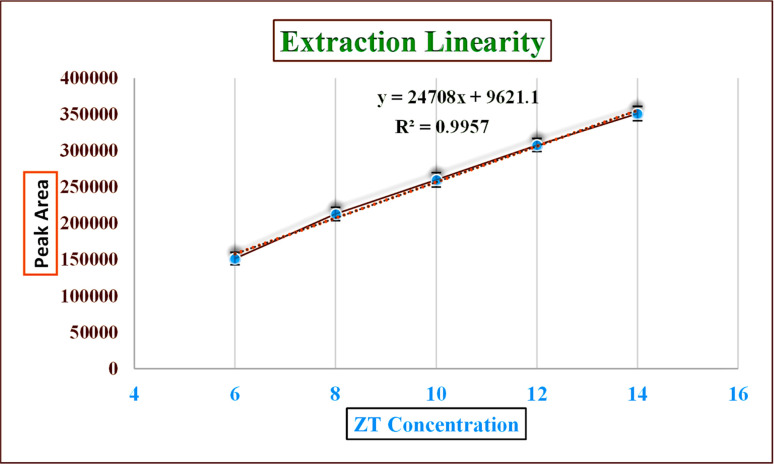
Extraction linearity of zolpidem from apple juice matrix in the 6 to 14 μg ml^−1^ concentration range.

From the standard linearity and extraction linearity plots, it could be seen that there was a decent association between the ZT concentration and the peak area, in the linearity range from the linear regression equation, as shown in Fig. 8 and 9 and chromatogram data in Tables S5 and S6.† Correlation coefficients (*R*^2^) of 0.998 and 0.9957 were determined, respectively, which are within ICH guidelines. The error bars depicted in Fig. 8 were calculated by determining the standard errors from three replicas of each linearity range concentration. For the LOD, and LOQ, signal to noise ratios of 3 : 1 and 10 : 1 were calculated, respectively, from the blank, giving values of 1.8 μg ml^−1^ for the LOD and 6 μg ml^−1^ for LOQ, whose chromatograms are shown in Table S7.†

##### 3.5.1.1. Adsorption capacity

The adsorption efficiency of PEI@ Fe_3_O_4_ NPs was assessed from the formula below ;^69^
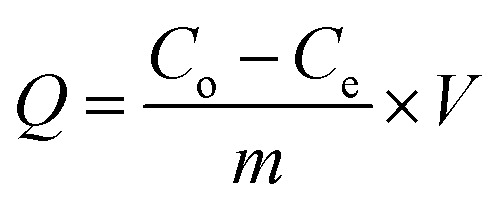
where *Q* is the adsorption capacity, *C*_0_ is the actual concentration of zolpidem, *C*_e_ is the concentration after DSPE extraction, *V* is the volume of sample, and *m* is the weight of nanoparticles in mg. The adsorption capacity was calculated from the actual concentration to that of the MNP-extracted ZT concentration in percentage form, in the 6–14 μg ml^−1^ concentration range. The adsorption capacity was found to be around 50% to 57%.

#### 3.5.2. Precision, accuracy, and robustness

Evaluation of the developed method's accuracy and precision was accomplished for a standard zolpidem tablet formulation. The inter-day and intra-day precision analyses were performed, resulting in percentage relative standard deviations (% RSDs) of 1.26, and 1.68 which are acceptable, as shown in Table 3 and by the chromatogram data in Table S9.† The % recovery and % RSD for precision and accuracy were also in the acceptable range as per ICH guidelines, as depicted in Tables 4 and 5, while the chromatogram data is in Table S8.† The robustness for mild variation did not show any marked differences in the resulting peak area, retention time, and tailing factor, confirming the newly optimized method was robust. Additionally, the method could be proven to be robust based on the % RSD obtained being below 1, *i.e.*, more preferable value, as calculated and shown in Table 6 and the chromatogram data in Table S10.†

**Table 4:** Precision of the method

Concentration (μg ml^−1^)	Inter-day precision		Intra-day precision	
	Conc. found (μg ml^−1^ ± SD)	% RSD	Conc. found (μg ml^−1^ ± SD)	% RSD
10 (100%)	11.31 ± 0.14	1.26	11.97 ± 0.20	1.68

**Table 5:** Accuracy of the method

Concentration (μg ml^−1^)	Conc. Found (μg Ml^−1^ ± SD)	% recovery	% RSD
5 (50%)	4.9 ± 0.04	102.43 ± 2.11	0.77
10 (100%)	10.25 ± 0.08	101.62 ± 4.2	0.70
15 (150%)	15.55 ± 0.02	102.22 ± 4.4	0.13

**Table 6:** Robustness of the method

Mobile phase composition variation						
Concentration		Results				
	Actual	Varied	Level of variation	Conc. found (μg ml^−1^ ± SD)	% RSD	
10 μg ml^−1^	60 : 40	66 : 34	+ 10%	11.24 ± 0.02	0.15	2.2
		60 : 40	0	11.34 ± 0.06	0.50	2.4
		54 : 46	−10%	11.12 ± 0.04	0.37	2.5

**Flow rate variation**						
10 μg ml^−1^	1 ml min^−1^	1.2 ml min^−1^	+ 0.2 units	10.09 ± 0.02	0.18	2.15
		1 ml min^−1^	0	11.34 ± 0.06	0.50	2.4
		0.8 ml min^−1^	−0.2 units	12.30 ± 0.08	0.63	2.6

**Wavelength variation**						
10 μg ml^−1^	294.6 nm	299.6 nm	+ 5 nm	11.24 ± 0.02	0.15	2.2
		294.6 nm	0	11.34 ± 0.06	0.50	2.4
		289.6 nm	−5 nm	11.12 ± 0.04	0.37	2.5

### 3.6. Green and white assessment

Evaluation of green and white principles was performed for the newly developed method for the identification and quantification of zolpidem tartrate, as portrayed in Table 7 and in comparison to previously reported methods, as shown in Table 8.

**Table 7:** Green and blue assessments for the current work for zolpidem detection

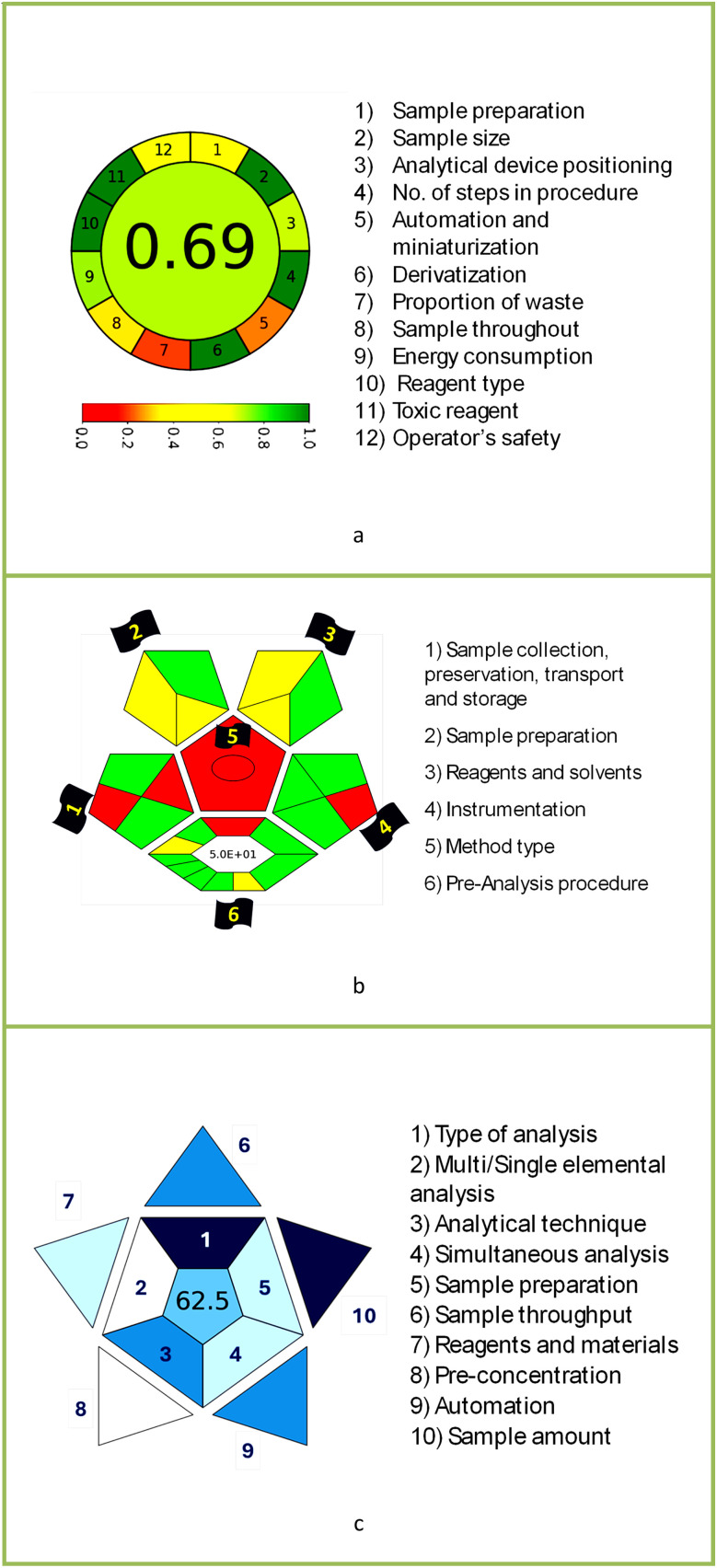

**Table 8:** Comparison of previously reported methods for the extraction and validation of zolpidem

Method description	AGREE	Complex-GAPI	BAGI
Thin film microextraction using LC-MS/MS^70^	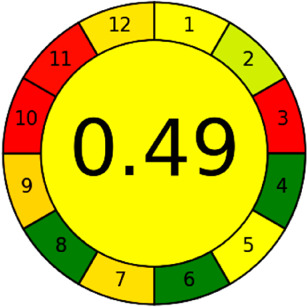	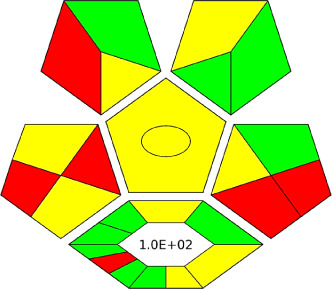	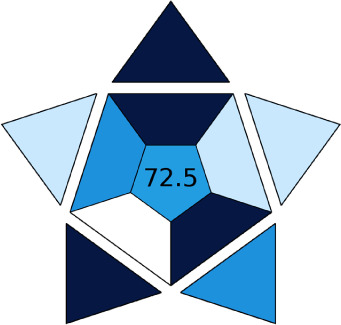
QuEChERS approach using LC-MS/MS and GC-MS/MS^71^	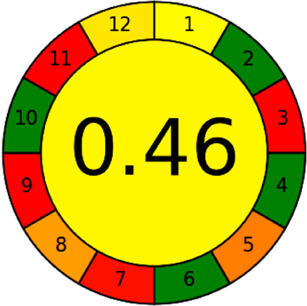	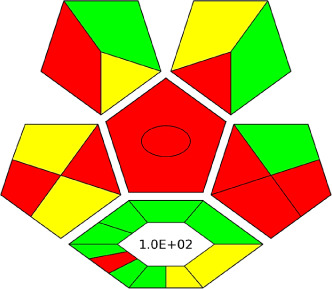	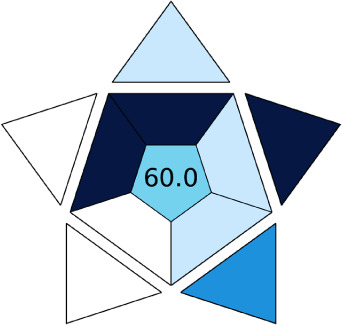
Polystyrene based SPE using LC-MS/MS^15^	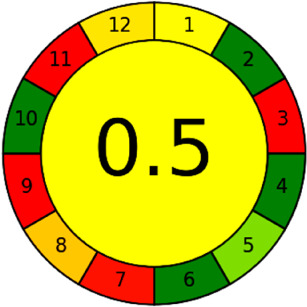	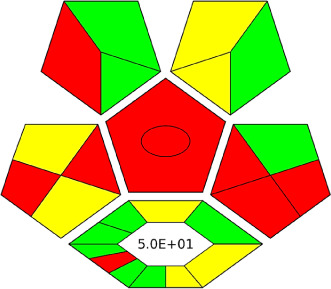	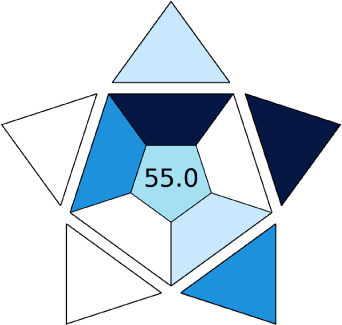
LLE using GC-MS/MS^72^	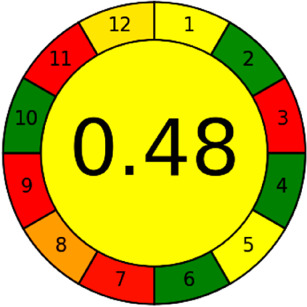	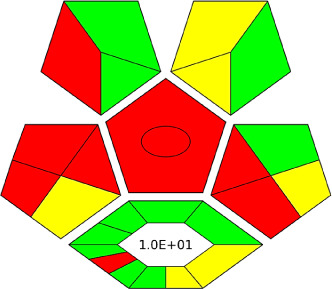	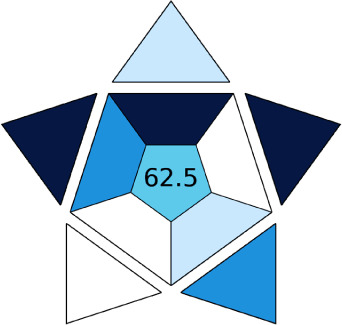
LLE using UPLC-MS/MS^73^	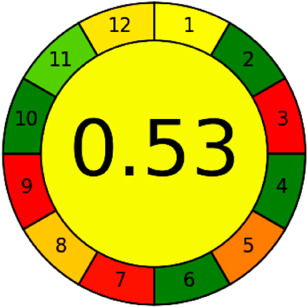	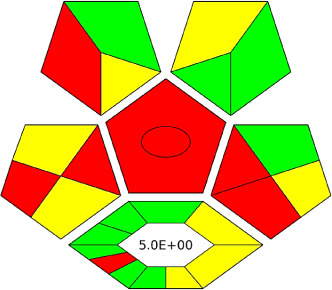	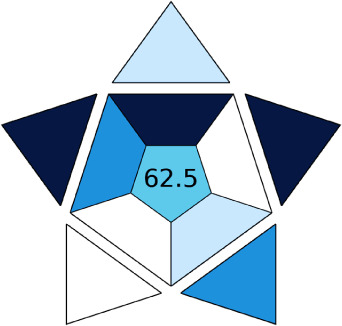
LLE based using UPLC-triple quadrupole^74^	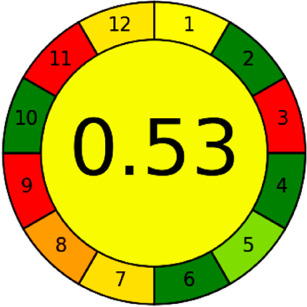	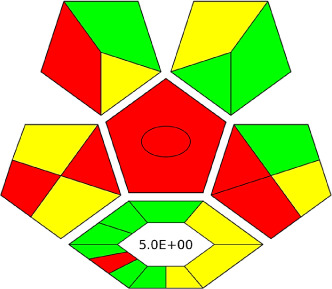	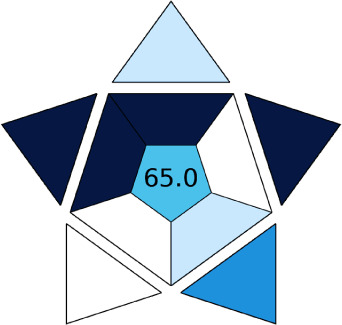
SPE using LC-MS/MS^75^	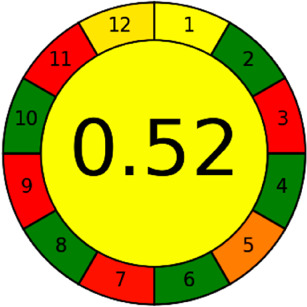	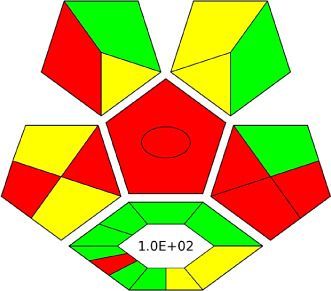	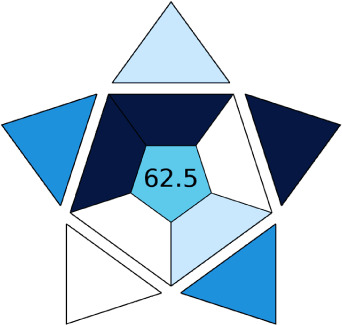

The AGREE software depicts the greenness of each of 12 Green Analytical Chemistry (GAC) principles in metric format, *i.e.*, between 0 to 1. As shown in Table 7a, the current method had an overall score of 0.69, reflecting the method's greenness as per the software, where an acceptable score is above 0.50. From Table 7a, the highest score of one was noticed with the number 2, 4, 6, 10, and 11 principles, highlighting the method's low energy consumption, minimal sample size, and omission of derivatizing or toxic reagents. A score of 0.6 was allotted to principles 3, 9, and 12, and a score of ∼0.5 for 1 and 8. The lowest scores were for 5 and 7.^48,76^ In Table 7b, the Complex-GAPI scores are given, which is a well referenced tool that can be used to easily describe a method's green nature by assessing the solvent/reagents used, amount of wastage, and consumption, along with a pre-analysis for comparison to allow appropriately interpreting the method's output. The pictogram for the current method showed more green shades due to the lack of storage, recyclability, slightly lower power consumption, and better NFPA score, while a few red shades could be observed due to the extraction procedure and waste production, which was unexceptional overall.^47^

BAGI is a primary tool that focuses on blue principles. It measures a method against applicability and practicality criteria, while displaying the result in an asteroid pictogram form along with a metric score. For the current method, a bluer compliance with a score of 62.5 was obtained, as shown in Table 7c, highlighting the method's economic, applicable, and practical potential using the common instrument HPLC, along with its quite good sample throughput, and low sample amount requirement. The need for a preconcentration step and nanoparticles lower the score, but these steps are common in SPE extraction procedures. Nevertheless, on the whole, the method reached the software criterion of a score higher than 60 to show the method has acceptable applicability.^50^

Comparing the current work to previously reported methods, the current work was proven to be greener. This was partly due to the reason that most of the reported methods use high-energy-requiring instrumentation, such as LC-MS/MS or GC-MS/MS or even both. Further, the use of harmful solvents, such as acetonitrile, chloroform, and hexane in these works is explicitly avoided in our current work. Also, most methods do not carry or involve recycling processes, which also benefits greenness. Further, energy conservation, recyclability, and the use of environmentally preferable solvents are a few of the 12 green principles, which were missing in previous methods that had scores below 0.5 (not green) or classed as a slightly greener method according to AGREE, as shown in Table 8. The same criteria exist for Complex-GAPI, where an extra red shade of color apart from the current method, was seen in many aspects, *i.e.*, in the energy consumption part, recycling, storage, and a few other criteria, for the previously reported methods, as clearly depicted in Table 8.

#### 3.6.1. RGBfast

This is the enhanced version of the previous RGB models,^77,78^ which, as an improvement, incorporates ChlorTox Scale as a part of assessing the green criteria and provides a comparative mode. Moreover, the latest version has automation and transparency, making the method more applicable, with a lower possibility of manipulation, and easy to operate.^49^

**Fig. 10:**
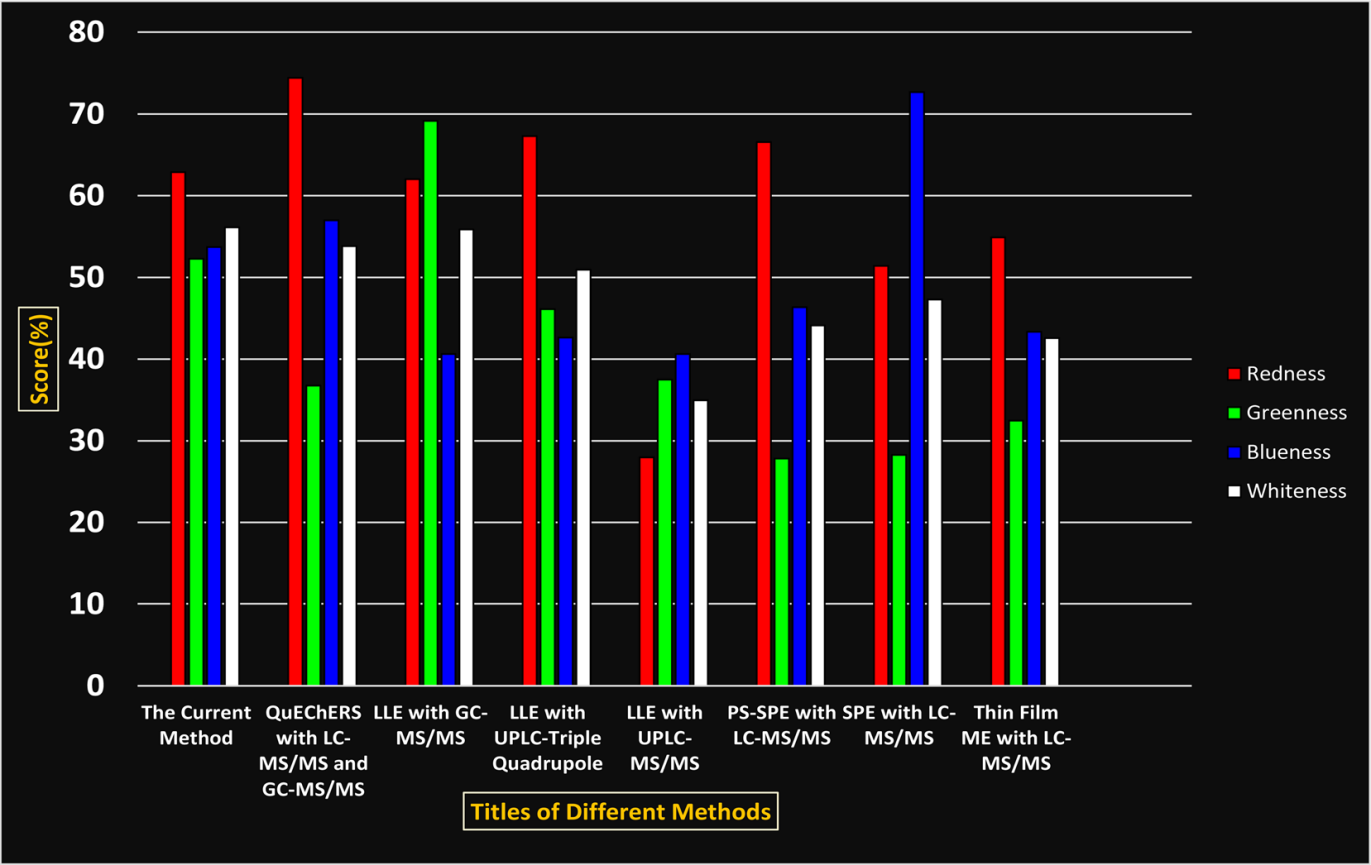
RGBfast comparative study.

**Fig. 11:**
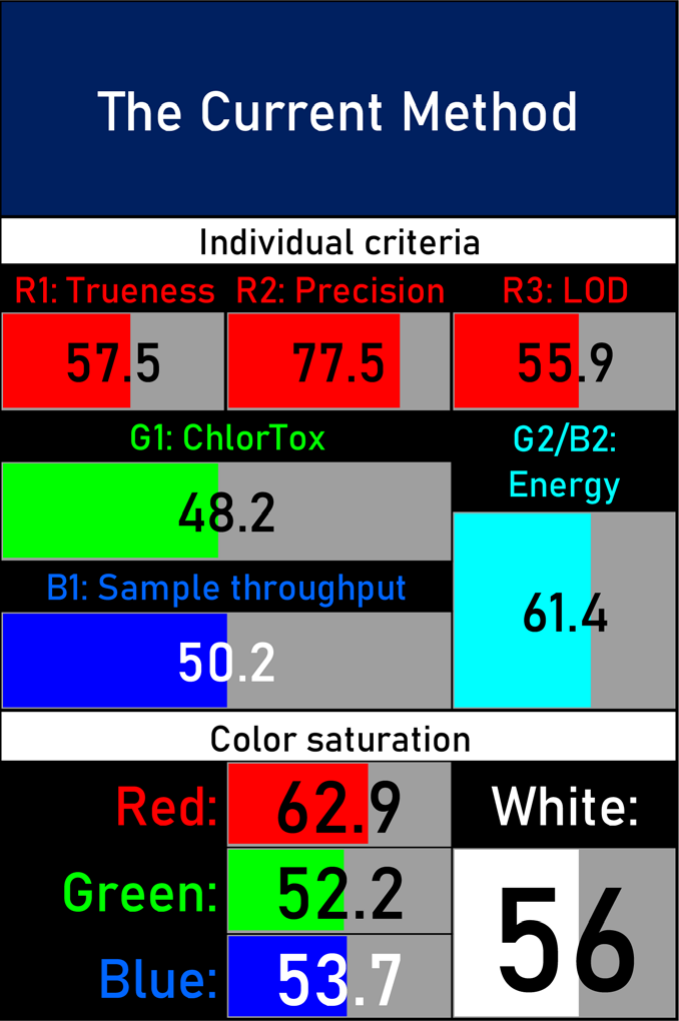
RGBfast model results for the current method.

A comparative study was performed between the current method and similar previous literature works. In this version, the balance is shown among the works in the comparative mode, rather than mentioning a particular work as greener or whiter. From the assessment, as shown in Fig. 10, there are few methods proving better green scores than others. The best green score was for ‘LLE with GC-MS/MS method’, as shown in Fig. S7b,† as it used a low solvent volume and instead used gas as the mobile phase, but it had a lower blue score because of its higher energy requirement. A high blue score was noticed in the SPE with LC-MS/MS method, as shown in Fig. S7f,† because of its comparatively higher sample throughput, whereas, it secured the lowest green score because of using more chemicals and solvents, especially for SPE preparation. A good red score was observed for the QuEChERS approach using the LC-MS/MS and GC-MS/MS method, as shown in Fig. S7a,† due to its better sensitivity, and other performance parameters, but it had a reduced green score due to the usage of both high energy consuming instruments and greater solvent consumption. Detailed scores for all the comparative methods for each principle are provided in the ESI, Fig. S7.† Now, approaching the current method, that has slightly increased red score to that of green, and blue score. Nevertheless, overall, a good white score (56%) has been obtained as in Fig. 11, which is a better score in comparison to other similar works.

### 3.7. LC-MS confirmation

The analysis resulted in a chromatogram that reflected RTs at 2.527 and 2.497 corresponding to the ZT tablet and ZT extraction from apple juice matrix. In the extraction chromatograms, there were very few matrix interferences noticed.

The mass spectra of both displayed a molecular ion peak at *m*/*z* 308 formed due to (*M* + 1) fragmentation. A further fragmentation eliminated the lateral groups, such as amide and carbonyl moiety,^79^ providing peaks at *m*/*z* 236 and 240. The complete chromatogram and MS spectra are shown in Fig. S2–S6,†12 and 13.

**Fig. 12:**
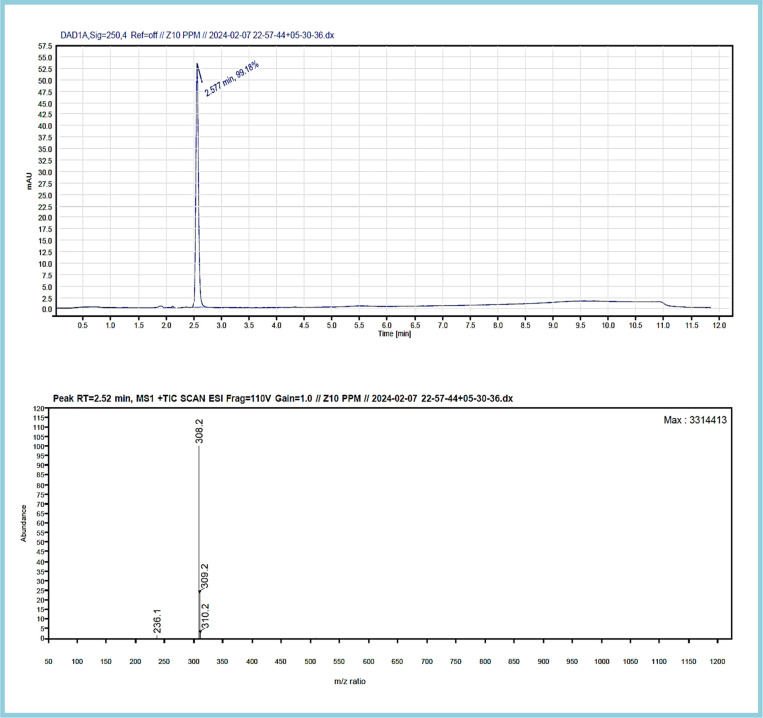
LC-MS chromatogram and mass spectrum of the zolpidem tablet formulation.

**Fig. 13:**
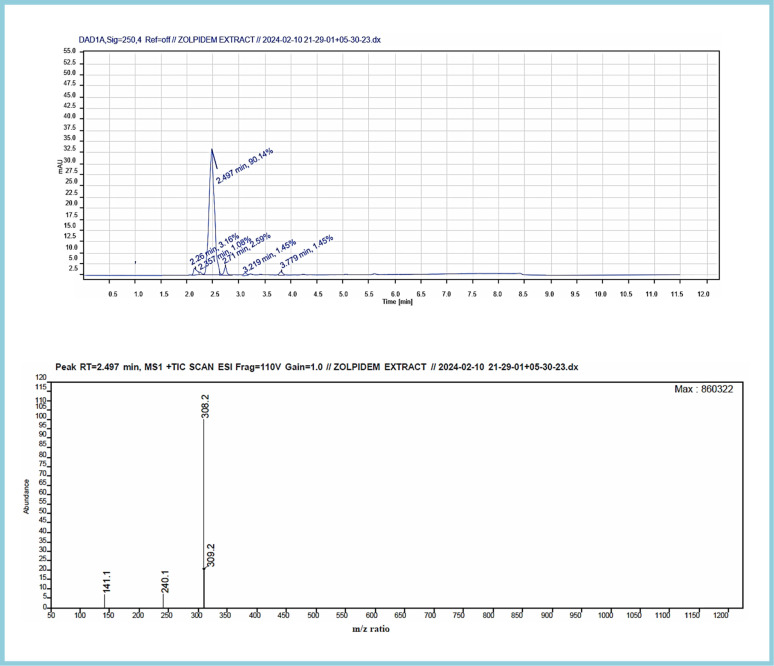
LC-MS chromatogram and mass spectrum of the extracted zolpidem from apple juice matrix.

## 4. Conclusion

In this research study, we successfully applied PEI@SiO_2_@Fe_3_O_4_ NPs for the extraction of zolpidem from apple juice matrix, for the first time. Moreover, a new method was optimized and validated for zolpidem, with the aid of RP-HPLC. The results obtained demonstrated the adequate sensitivity, robustness, accuracy, and linearity of the proposed method, as the values were within the ICH guidelines, as also evidenced through the RGBfast model for a red assessment. Ultimately, for better resolution, accurate confirmation, and better understanding of the matrix presence, LC-MS was also performed and proved the presence of zolpidem from the extraction through the mass spectrum, while the effect of the matrix was minimal with good resolution in the LC chromatogram. The greenness and whiteness of the newly proposed method were ascertained by the Complex-GAPI system, resulting in more greener shades, while AGREE software was with a good green score of 0.68 in green assessment. Meanwhile, the BAGI score of 62.5% under consideration of the blue principle showed the methods' practicality and applicability. Ultimately, a white score of 56% was secured, *via* the RGBfast model, suggesting the method offered a quiet good balance of all three sustainability principles (Red, Green, and Blue). Additionally, these assessments were carried in comparison with other previously reported recent methods for zolpidem detection, which explicitly proved our current method to be better under green principle considerations and offered an overall good balance of RGB principles. Henceforth, further the current work can be applied for other types of fruit juice matrices, as the extraction procedure includes TFA, which can degrade sugars and proteins easily. Besides this, other drugs related to crime and abuse in different edible matrices can also be detected as a future potential application scope for the method.

## Data availability

The authors confirm that the necessary data are provided in the ESI file† for the related work. Further information or raw data, if required, will be provided upon a reasonable request to the corresponding author.

## Author contributions

Revathy Sundara Moorthy – conceptualization, investigation, formal analysis, methodology, validation, software, data curation, writing – original draft, resources. G. Swetha – conceptualization, supervision. Anren Hu – funding acquisition. P. Muralidhar Reddy & Rohini Rondla – supervision, project administration, writing – review & editing, resources. Narmada Vallakeerthi – resource.

## Conflicts of interest

There are no conflicts to declare.

## Supplementary Material

RA-014-D4RA04303K-s001
