# Supplementary material for: Greener and whiter analytical method development and validation for determining the presence of zolpidem tartrate infused in apple juice using RP-HPLC via magnetic solid-phase extraction followed by LC-MS confirmatory analysis
Source: RSC Adv. 2024 Sep 4;14(38):28168–81. doi: 10.1039/d4ra04303k (PMC11372563; doi:10.1039/d4ra04303k)
Supplement: RA-014-D4RA04303K-s001 [file RA-014-D4RA04303K-s001.pdf]

Supplementary Information (SI) for RSC Advances.
This journal is © The Royal Society of Chemistry 2024

Greener and Whiter Analytical Method Development and Validation of Zolpidem Tartrate Infused in Apple Juice using RP-HPLC via Magnetic Solid Phase Extraction followed by LC-MS confirmatory analysis

Revathy Sundara Moorthy[a], G. Swetha[a], Rohini Rondla[b], Anren Hu[*c], Narmada Vallakeerthi[d] and P. Muralidhar Reddy[*a]

Supplementary data

Preparation of 0.25 mg/ml ZT stock solution

It was prepared by weighing 6.25 mg Active Pharmaceutical Ingredients (API) of ZT and transferred into 25ml standard solution. The drug was filled to the mark of standard flask using distilled water

Preparation of 100µg/ml standard solution.

100µg/ml standard solution was prepared by weighing 10mg of ZT API and transferring into 100ml standard flask. The flask was made to the mark using methanol.

Optimization of different parameters

Figure S1: Zolpidem API standard chromatogram

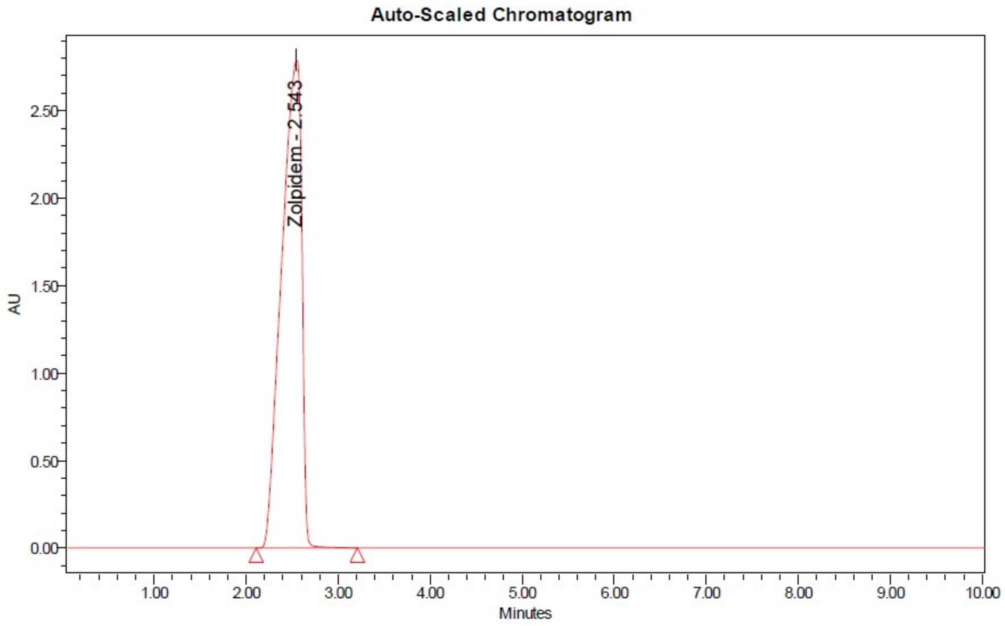

Characterization

Table S2: FTIR characterization of Fe_3O_4, SiO_2@Fe_3O_4, APTES@Fe_3O_4, PEI@SiO_2@Fe_3O_4 NPs

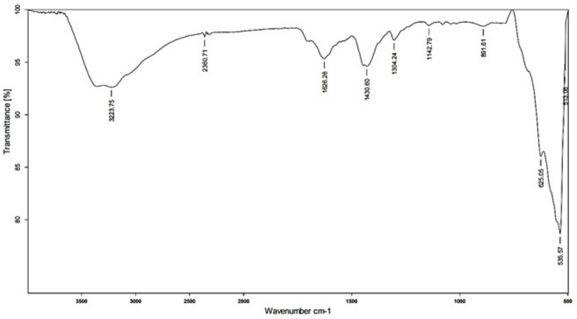

Fe_3O_4

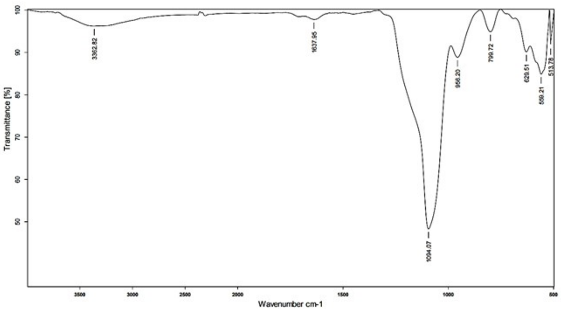

Fe_3O_4@SiO_2

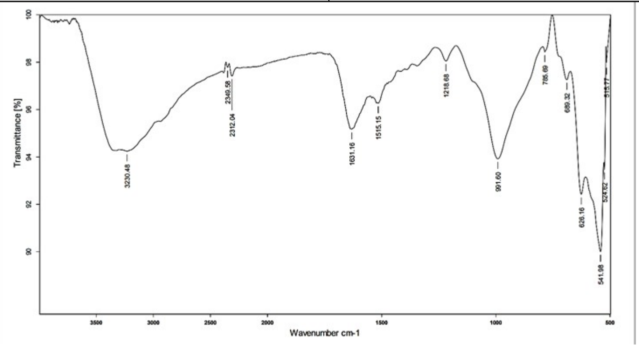

Fe_3O_4@APTES@PEI

Table S3: SEM-EDX of Fe_3O_4, SiO_2@Fe_3O_4, APTES@Fe_3O_4, PEI@SiO_2@Fe_3O_4 NPs

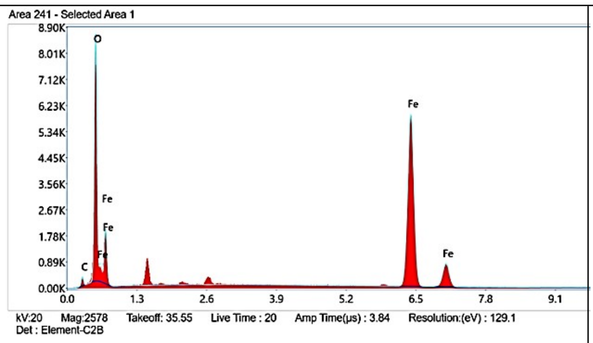

Fe_3O_4

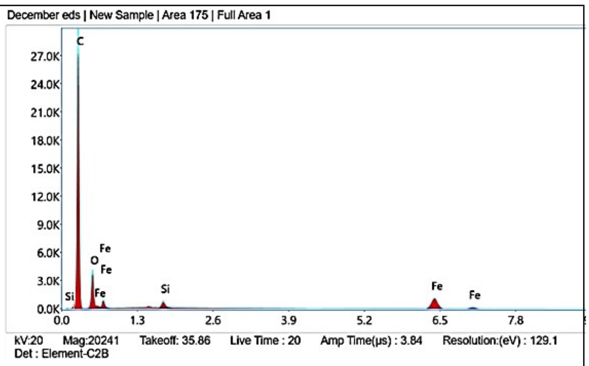

Fe_3O_4@SiO_2

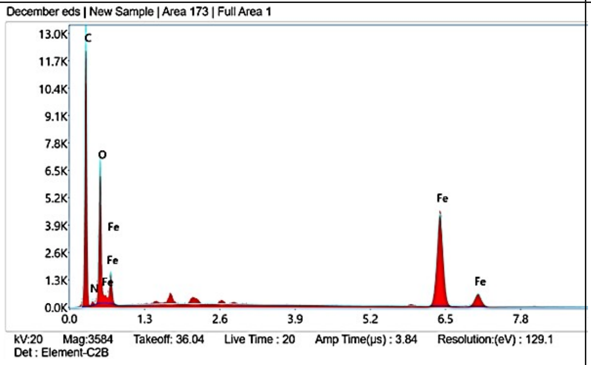

Fe_3O_4@PEI

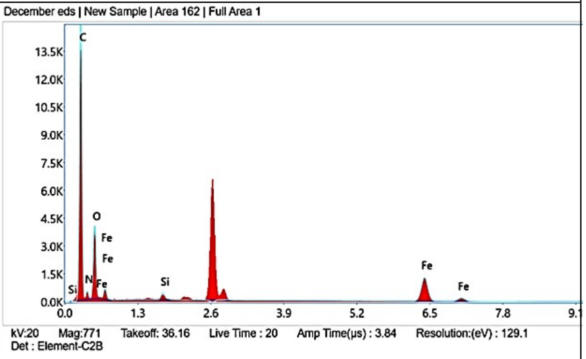

Fe_3O_4@APTES

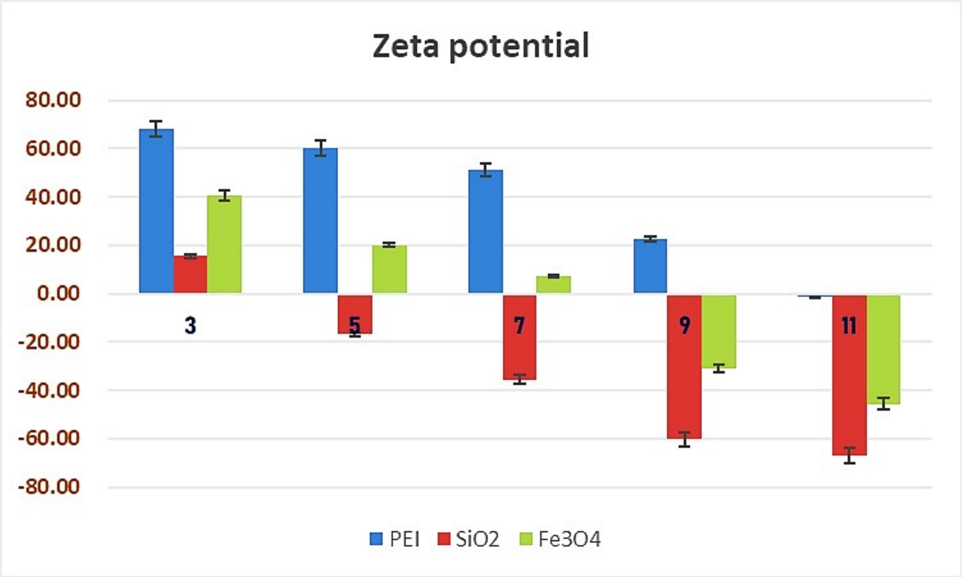

Figure S1: Zeta potential of Fe_3O_4, $SiO_2@Fe_3O_4$, APTES@Fe_3O_4, PEI@$SiO_2@Fe_3O_4$ NPs

<u>Morphological characterization</u>

Table S4. SEM and TEM images of Fe_3O_4, $Fe_3O_4@SiO_2$ NPs, $Fe_3O_4@SiO_2$@PEI NPs

**SEM**	**TEM**
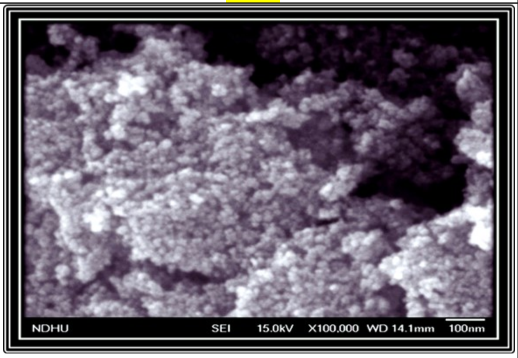 Fe_3O_4 NPs	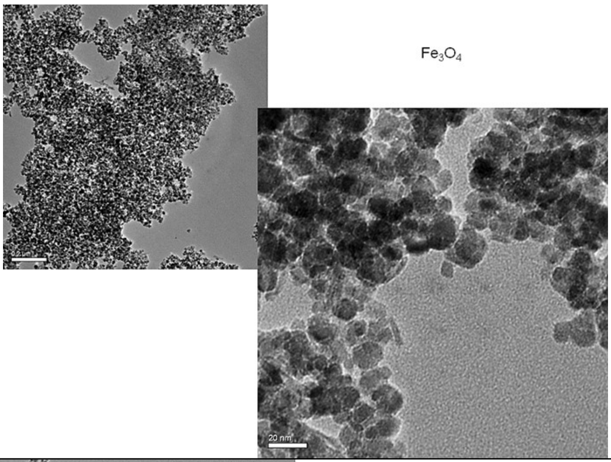
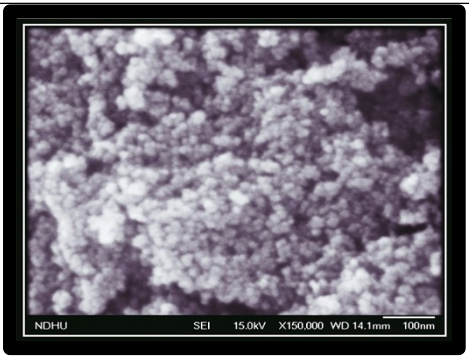 $Fe_3O_4@SiO_2$ NPs	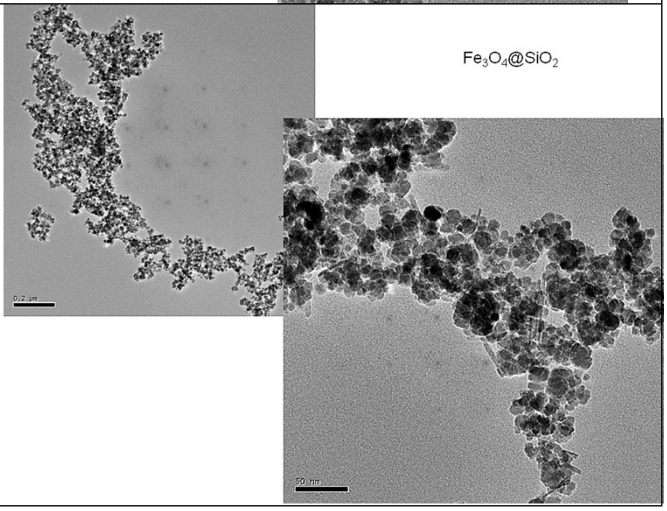

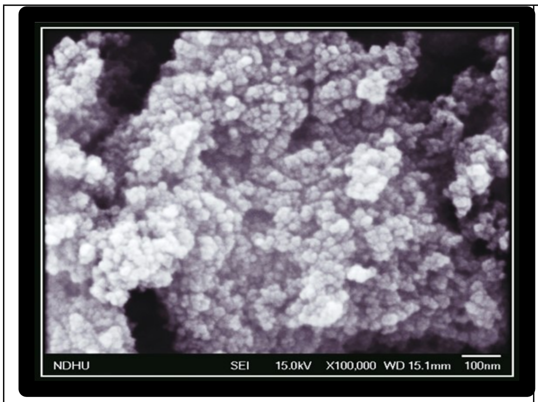

$Fe_3O_4@SiO_2@PEI$ NPs

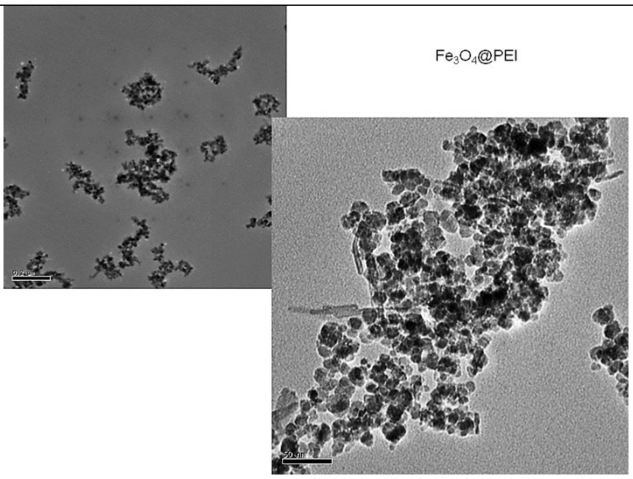

$Fe_3O_4@SiO_2@PEI$ NPs

Method Validation

Table S5: Zolpidem tablet Standard Linearity Chromatograms from 6 to 14 ppm

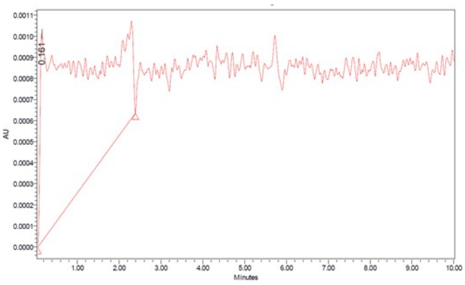

Blank

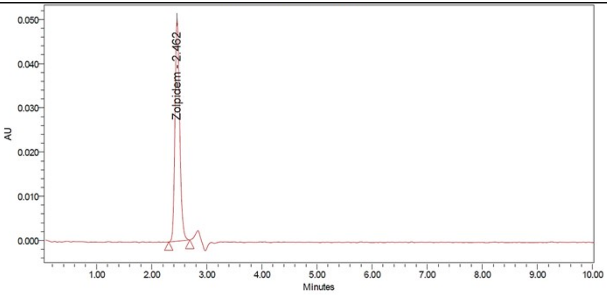

6PPM

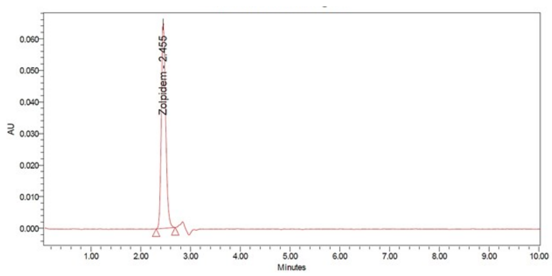

8PPM

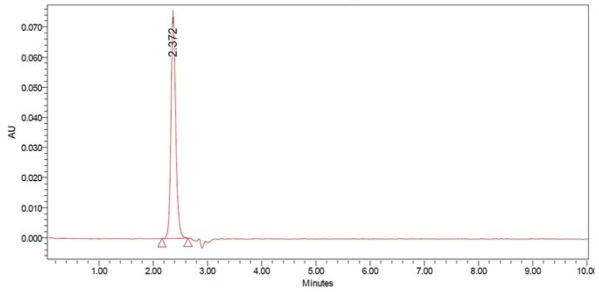

10 PPM

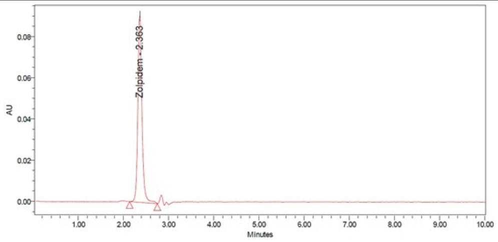

12 PPM

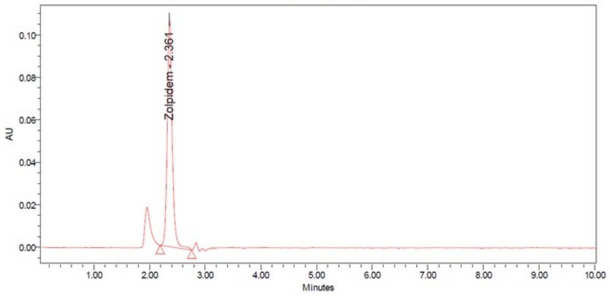

14 PPM

Table S6: Zolpidem extraction linearity Chromatograms from 6 to 14 ppm

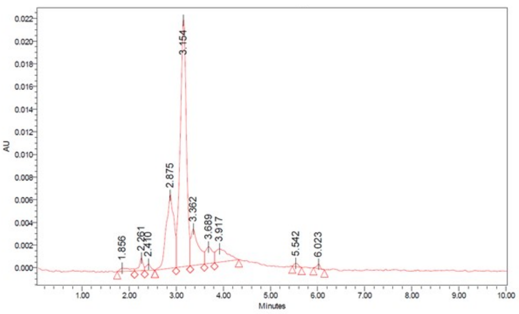 Extraction Blank	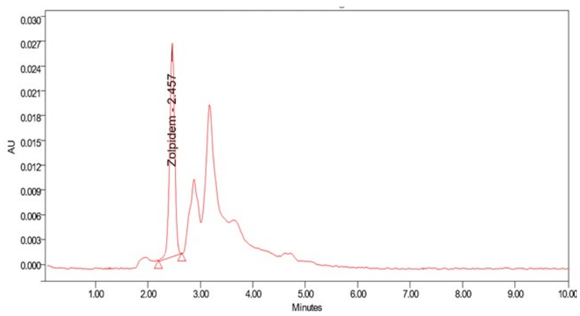 6PPM
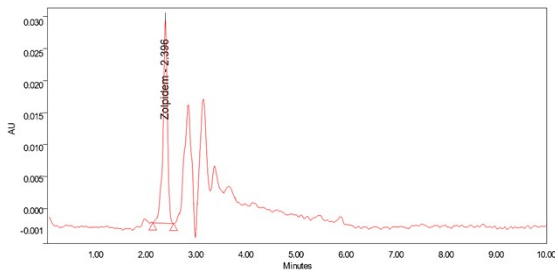 8 PPM	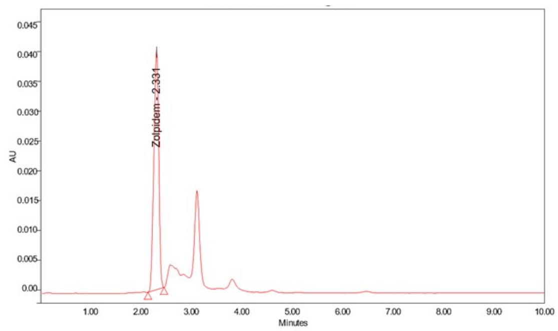 10PPM
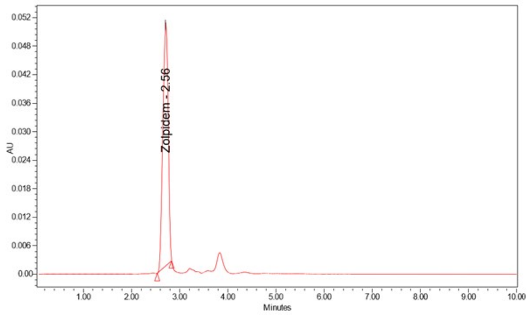 12 PPM	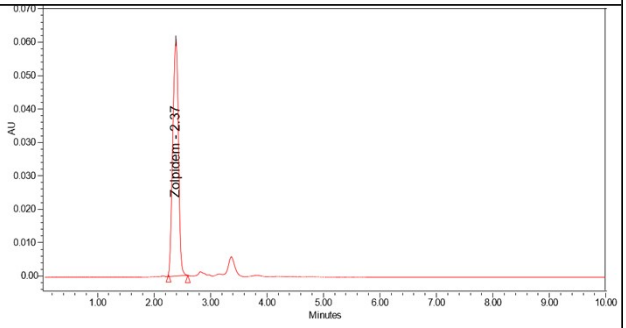 14PPM

Table S7 Chromatograms of LOD and LOQ

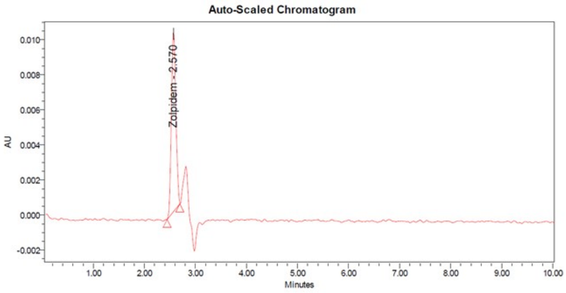 LOD	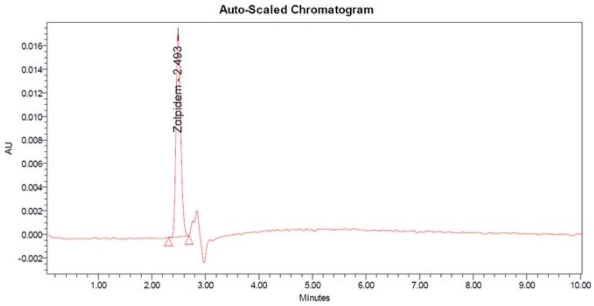 LOQ

Table S8: Accuracy chromatograms

Accuracy 50%

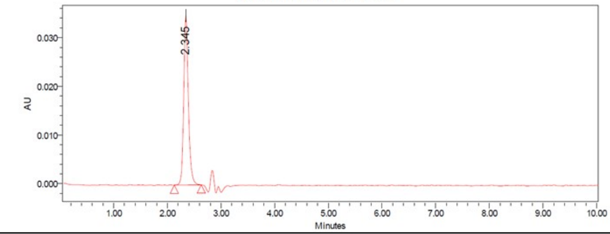

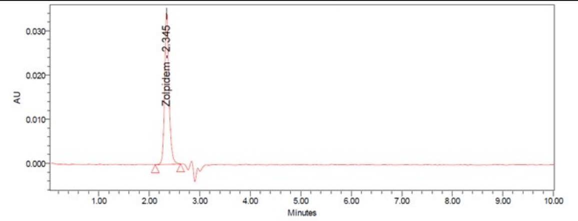

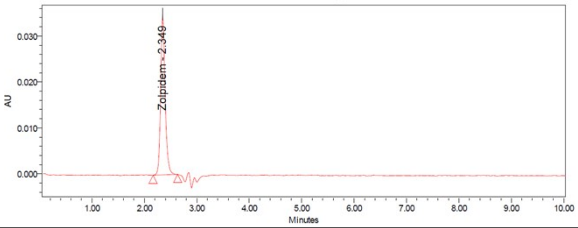

Accuracy 100%

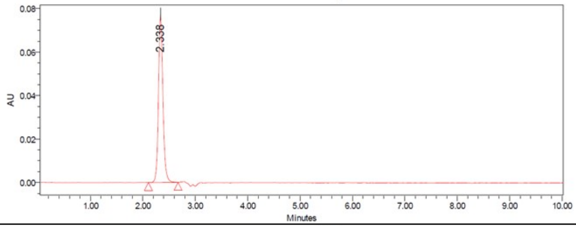

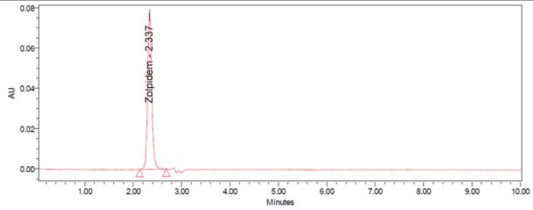

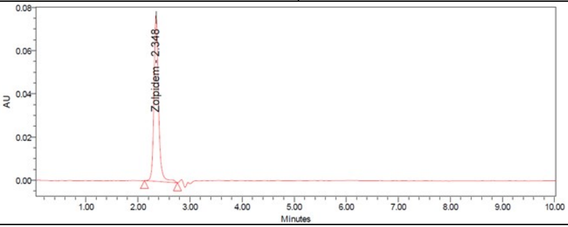

Accuracy 150%

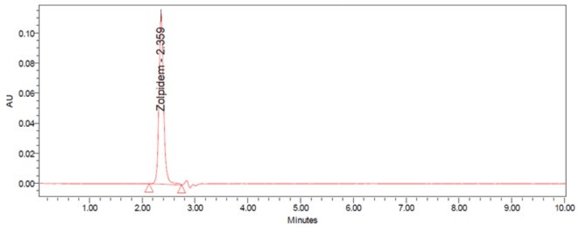

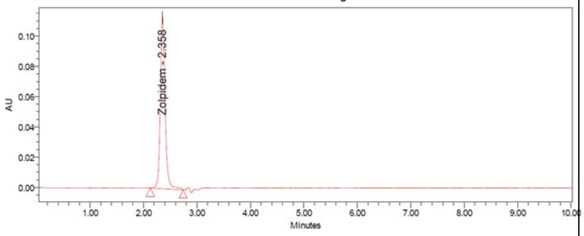

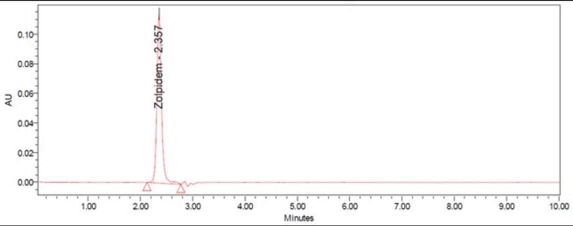

Table S9: Precision for chromatograms

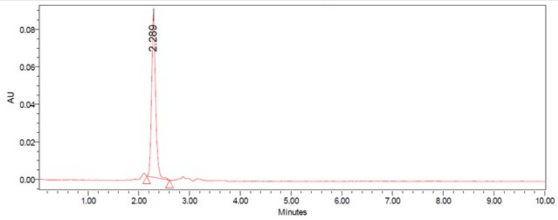

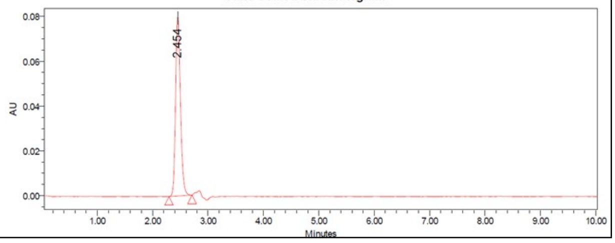

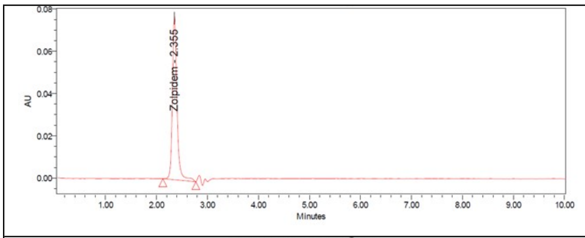	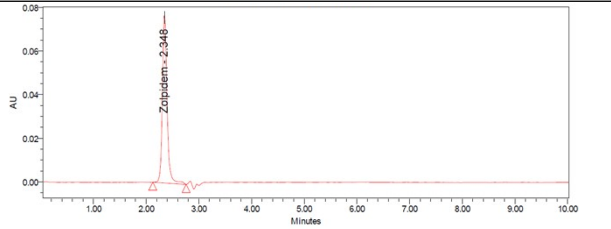
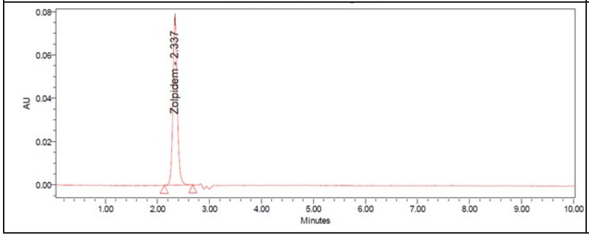	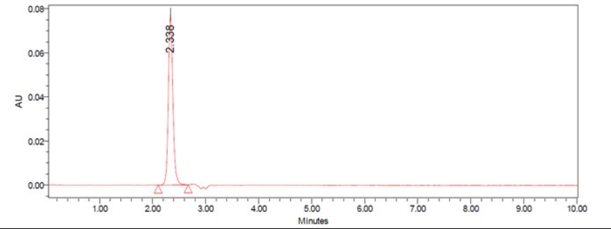

Table S10: Robustness chromatograms

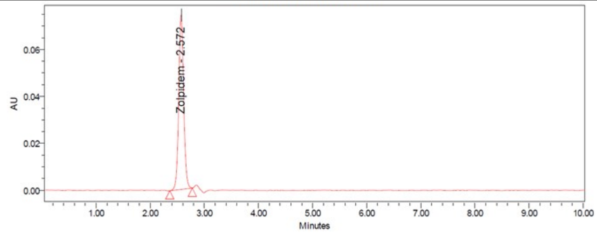	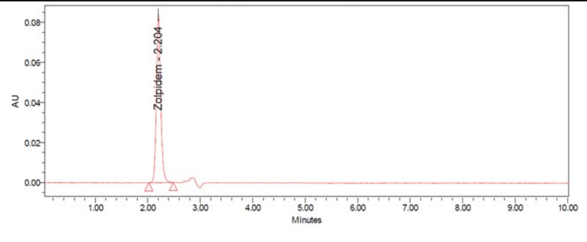
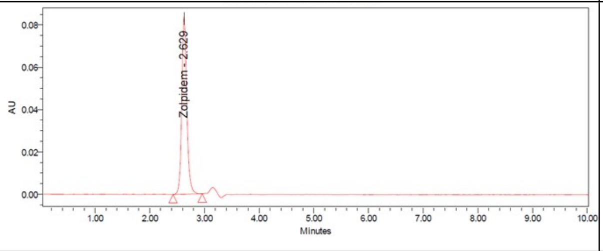	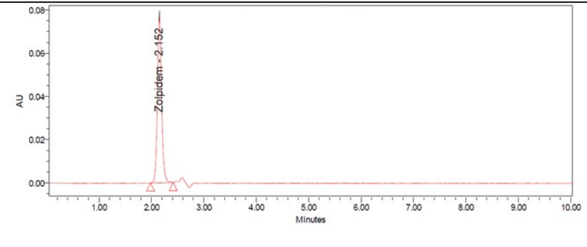
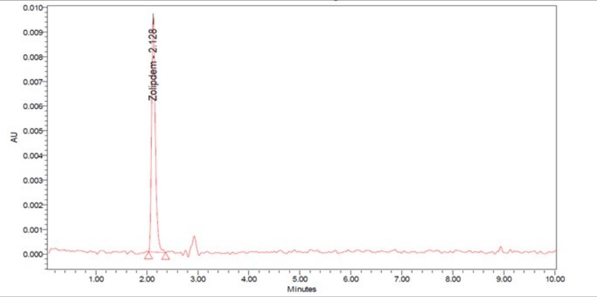	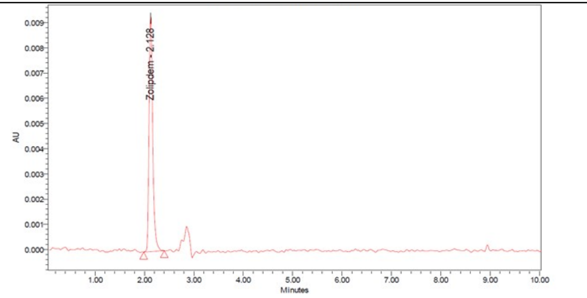

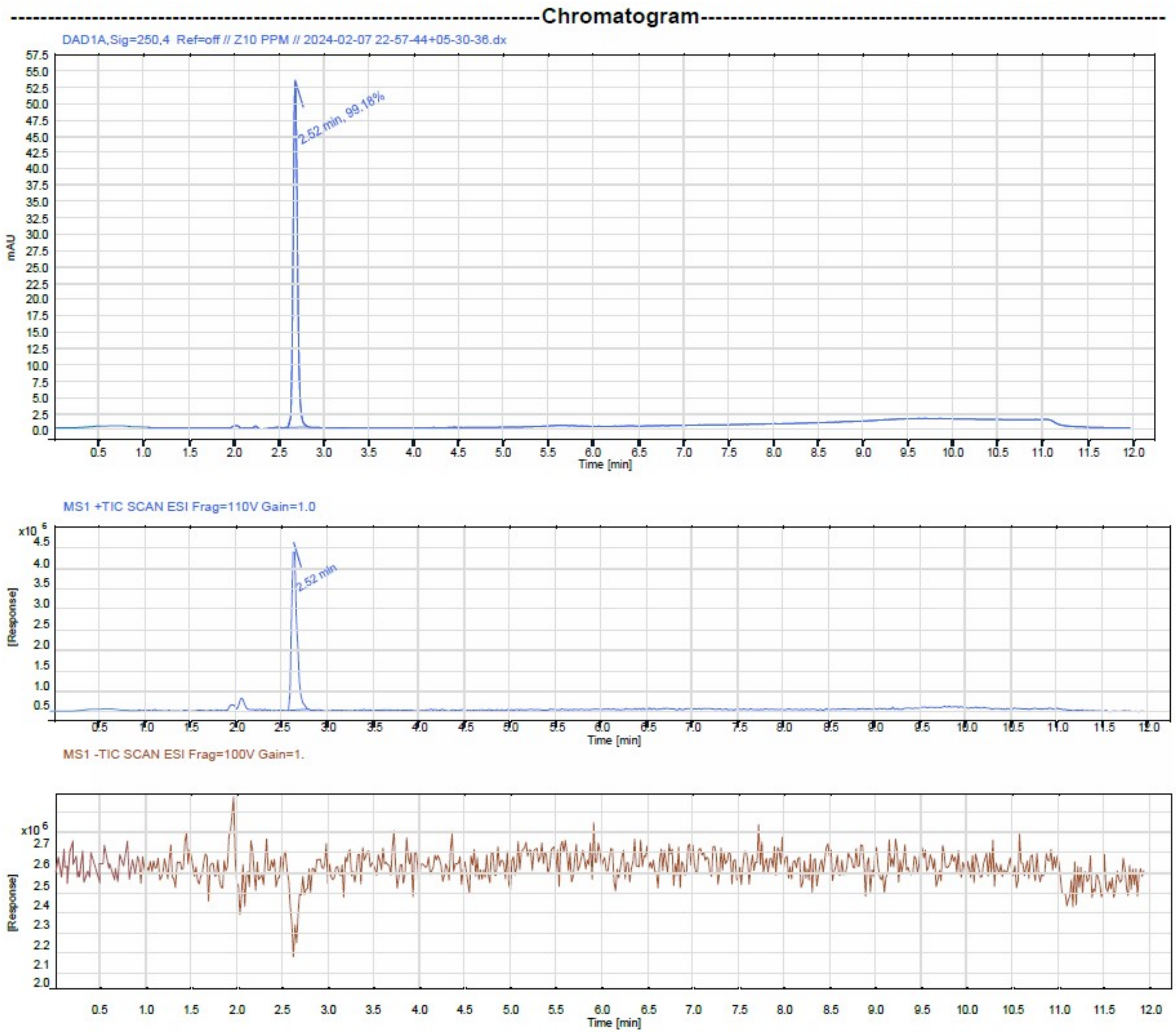

Figure S2: Zolpidem Standard LC-MS chromatogram

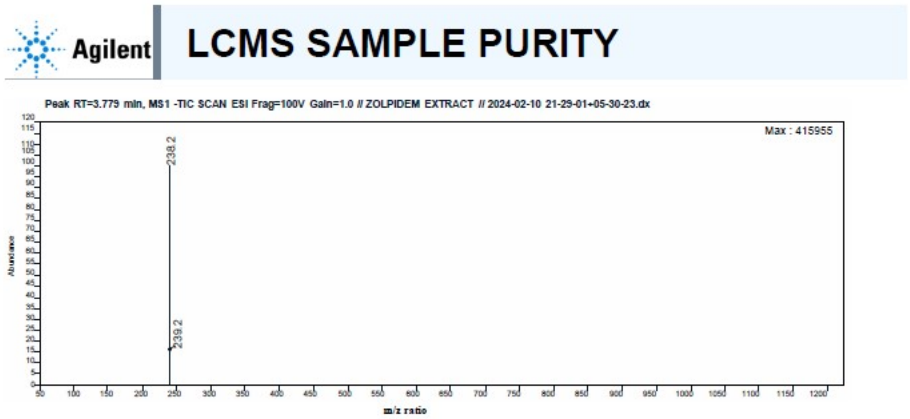

Figure S3: Zolpidem Standard Mass Spectrum

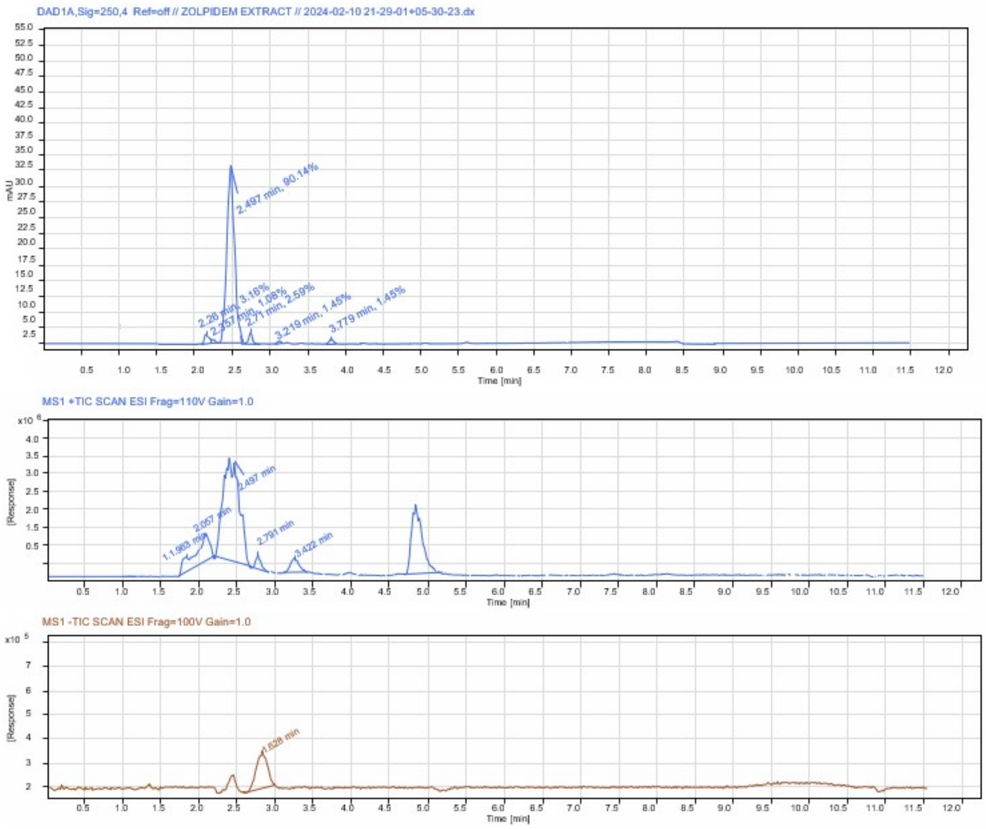

Figure S4: Zolpidem Extract from Apple juice matrix LC-MS chromatogram

LCMS SAMPLE PURITY

RT [min]	Area	Height	Area%
2.263	74.2797	2.7208	3.1589
2.357	25.4187	1.3207	1.0810
2.497	1119.6759	32.5345	90.1423
2.719	60.8122	2.6228	2.5861
3.219	15.3546	0.8394	0.6530
3.766	7.7700	1.3217	0.3304
3.072	3.5885	0.6783	0.1526
3.779	34.1311	1.9240	1.4515
4.405	5.7159	0.2578	0.2431
5.745	4.7311	0.1693	0.2012

--MS Spectrum--

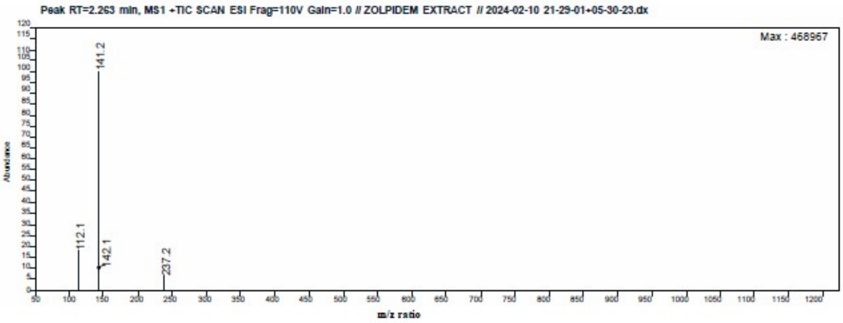

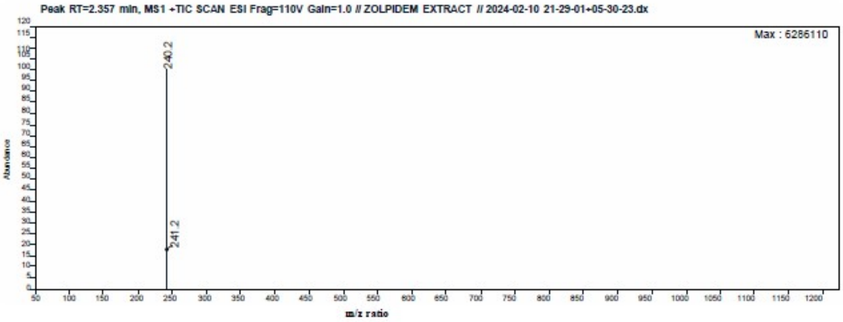

Figure S5: Matrix Mass Spectrum

Agilent

LCMS SAMPLE PURITY

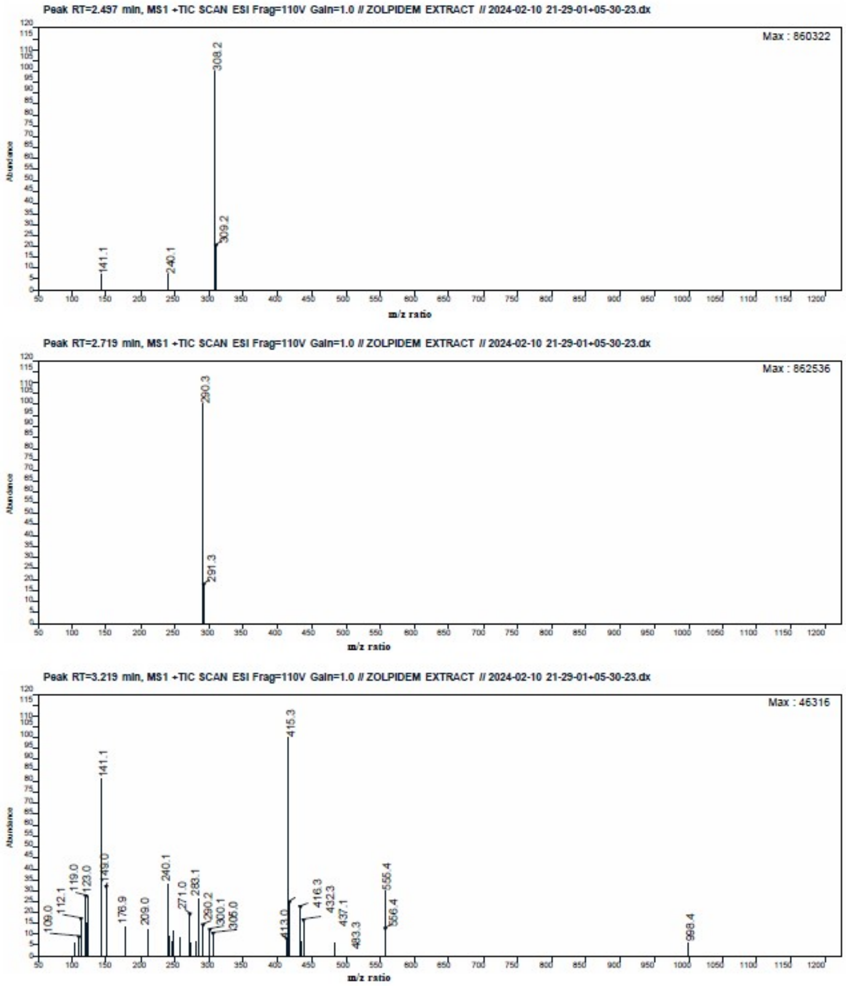

Figure S6: Zolpidem and Matrix Mass Spectrum

RGBfast

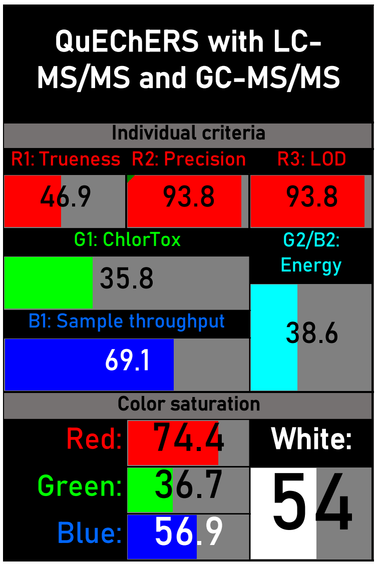

a

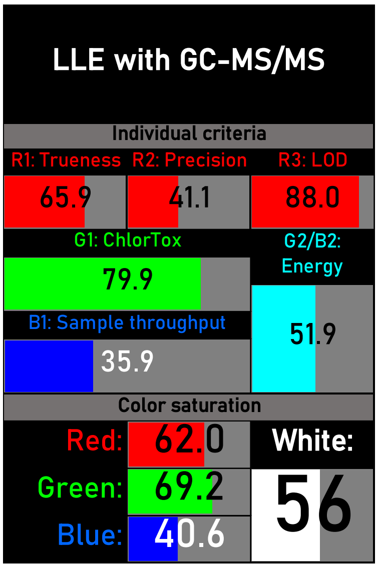

b

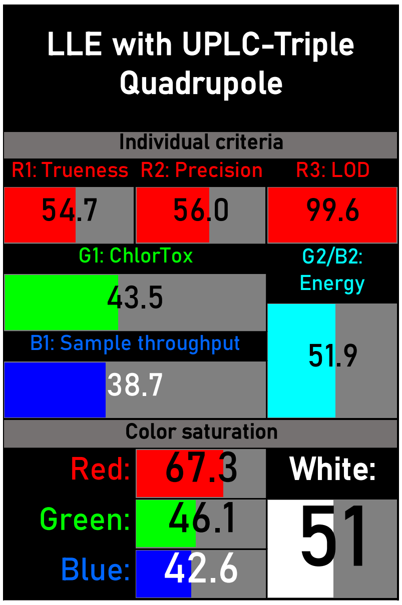

c

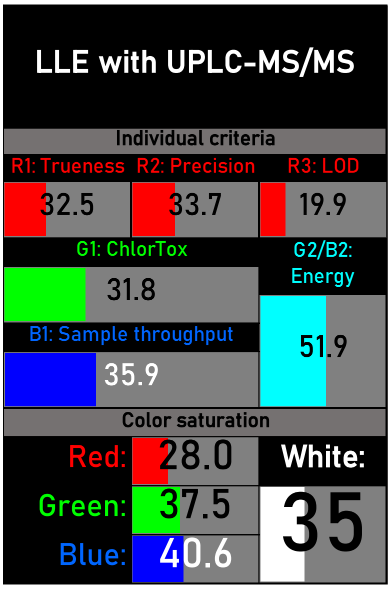

d

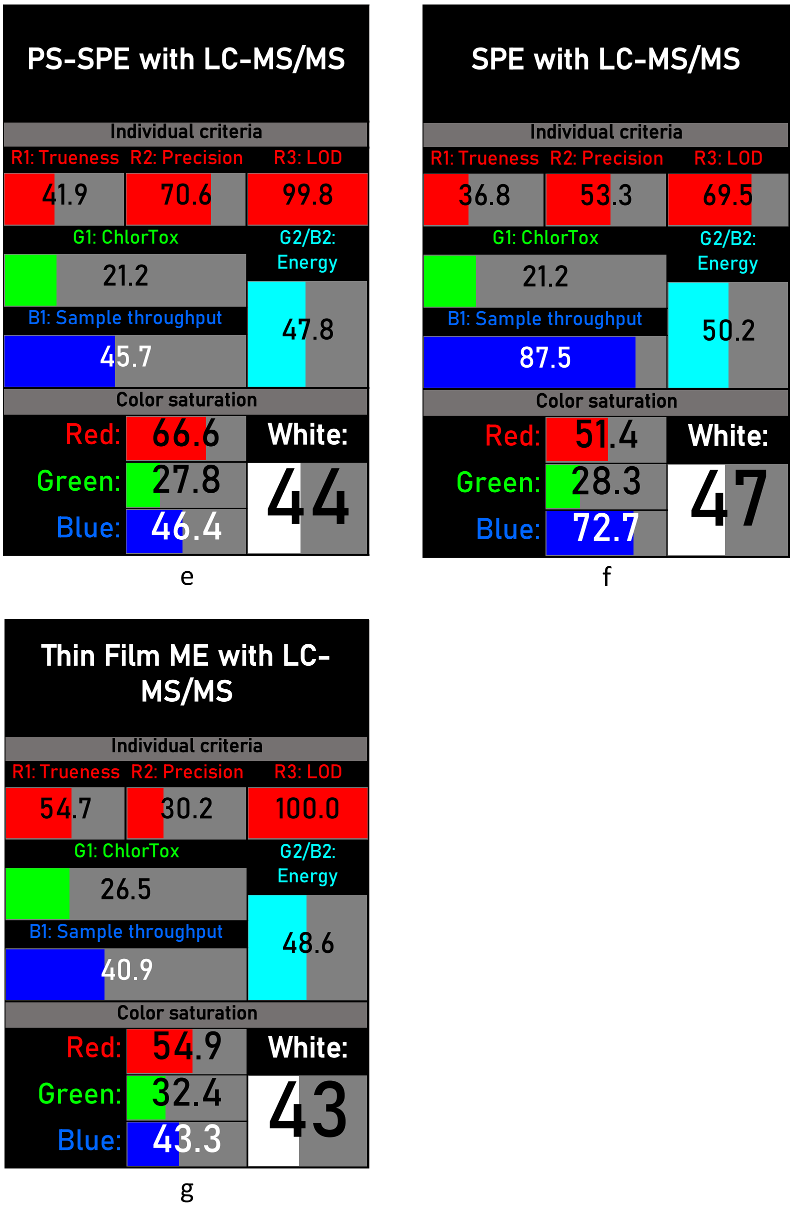

Figure S7: Comparative study of previous similar methods with detailed RGBfast
